# Supplementary material for: Stable isotopes show Homo sapiens dispersed into cold steppes ~45,000 years ago at Ilsenhöhle in Ranis, Germany
Source: Nat Ecol Evol. 2024 Jan 31;8(3):578–88. doi: 10.1038/s41559-023-02318-z (PMC10927559; doi:10.1038/s41559-023-02318-z)
Supplement: Supplementary file 1 — Supplementary Texts 1–5, Figs. 1–15 and Tables 1–6. [file 41559_2023_2318_MOESM1_ESM.pdf]

nature ecology & evolution

Article https://doi.org/10.1038/s41559-023-02318-z

Stable isotopes show *Homo sapiens* dispersed into cold steppes ~45,000 years ago at Ilsenhöhle in Ranis, Germany

In the format provided by the authors and unedited

Table of contents

1 Site description **2**
1.1 Excavations and stratigraphy of the lower sequence 2

2 Oxygen stable isotope palaeoclimatology **6**
2.1 Oxygen palaeoclimatology background 6
2.2 Hydrotopographical setting and non-temperature effects on oxygen stable isotopes 10

3 Strontium isotopes and animal spatial ecology **13**

4 Zinc stable isotopes **16**
4.1 Principles of zinc stable isotope variability 16
4.2 Impacts of bedrock lithology on zinc stable isotopes in Ranis 18
4.3 Zinc stable isotopes and herbivore feeding ecology 20

5 Extended methods **23**
5.1 Tooth enamel sampling 23
5.1.1 Sequential powder sampling 23
5.1.2 Bulk piece sampling 24
5.2 Oxygen stable isotope analysis 24
5.2.1 Silver phosphate preparation 24
5.2.2 Oxygen stable isotope measurements 25
5.3 Zinc and strontium stable isotope analysis 27
5.4 Collagen extraction and radiocarbon dating 29
5.5 Carbon and nitrogen stable isotope analysis 31
5.6 Inverse modelling 31
5.7 Palaeotemperature estimation 33
5.8 Software and code 35

6 Supplementary Figures **35**

7 Supplementary Tables **51**

References **69**

Other Supplementary Material for this manuscript includes the following:

Data and code to reproduce the manuscript files, figures and analyses are available at https://osf.io/wunfd/

1 Site description

1.1 Excavations and stratigraphy of the lower sequence

Ilsenhöhle in Ranis (50°39.7563'N, 11°33.9139'E, hereafter: Ranis) is a cave site located in the Orla river valley close to the city of Pößneck in eastern Thuringia, central Germany (Extended Data Figure 1). The cave system originally consisted of a large chamber with a south-facing entrance and two side chambers in a Permian limestone reef, but several roof collapses during the Pleistocene have left only two short sections of the side chambers preserved to the modern day. Above the cave, the top of the limestone rock houses a Medieval hill castle, Burg Ranis, built in the 11th century. Several excavation campaigns were conducted at Ranis in the 1920s and 1930s, first by then-owner of Burg Ranis, Dietrich von Breitenbuch, in 1926, 1929, 1931 and 1932, followed by Werner Hülle from the archaeological state office in Halle (Saale) who extensively excavated from 1932–1938[1]. Finally, re-excavations to document the site using modern excavation methods followed from 2016–2022 by the Thuringian State Office for the Preservation of Historical Monuments and Archaeology (TLDA) and the Department of Human Evolution, Max-Planck-Institute for Evolutionary Anthropology, Leipzig (MPI-EVA).

The early excavations by von Breitenbuch and Hülle covered more than 200 m^2 and removed most of the in-situ deposits including all material in the two side chambers and large parts of what would have been the main chamber[1]. Uncovered deposits included Medieval, Iron Age, Mesolithic, Upper Palaeolithic, transitional, and Middle Palaeolithic layers. The Palaeolithic archaeological layers were numbered III to XI and described as covering an age range from the Middle Palaeolithic to the Late Glacial. Relevant here is the lower part of the Pleistocene sequence including Layer VII Upper Brown Layer ("Obere Braune Schicht"), Layer VIII Black Layer ("Schwarze Fundschicht"), Layer IX Middle Brown Layer ("Mittlere Braune Schicht"), Layer X Grey

Layer (“Graue Schicht”), and Layer XI Lower Brown Layer (“Untere Braune Schicht”) (Extended Data Figure 1). Of particular interest to this study is the Grey Layer (Layer X, “Graue Schicht”) in the lower part of the sequence, which yielded an assemblage of 23 bifacial leaf points and 17 Jerzmanowice blade points, subsequently used to define the ‘Ranisian’ as part of the Middle to Upper Palaeolithic transitional technocomplex of the Lincombian-Ranisian-Jerzmanowician (LRJ)[2,3].

The excavation area of the 1930s was divided into 1 x 1 m squares and finds recorded accordingly with square and approximate depth information, but excavations were conducted using heavy duty tools and finds were mostly pooled into rough stratigraphic units without regular depth recording for individual specimens. While this is not uncommon for excavations in the 1930s, it poses challenges for assigning stratigraphic context of finds and the faunal collection additionally suffers from a lack of clarity and inconsistencies in the context documentation of finds. Boxes of faunal remains were commonly labelled with square, layer colour and approximate depth extent of the layer but without the layer numbering system that seems to have been implemented after excavations had been completed. Unfortunately, this often makes layer assignments very challenging, as different layers can have the same colour description (e.g., “braun” meaning “brown”). Conversely, different colour descriptions (i.e., chocolate brown, red brown) have been used for what appears to refer to the same stratigraphic unit, likely representing natural colour changes between different areas of the site. Further, depth labels are in many cases of limited utility in assigning stratigraphic provenance due to the sloped deposits and compression of layers due to rock fall in some areas. Finally, a number of inconsistencies in the documentation of stratigraphic provenance have been documented with mismatches between box labels, notes in the site monograph and original excavation documentation kept in the Museum für Vorgeschichte in Halle[4]. Clearly, the documentation of the collection suffered substantially from the historical events accompanying its curation, with original documentation lost in part, an in-

ability of W. Hülle to revisit the collection while writing the publication manuscript and finally his unexpected death culminating in a posthumous publication that is not always consistent with his original excavation diaries[4].

The need to clarify the stratigraphy of this important site and establish the makers of the LRJ prompted a re-excavation of the site in a small area to the southwest of the earlier excavations conducted by the TLDA/MPI-EVA starting in 2016[5]. The excavation area was placed immediately adjacent to and partially overlapping with trenches from the Hülle excavation and established a more detailed and more subdivided, but clearly correlated, stratigraphic sequence[5] (Extended Data Figure 1). In this new TLDA/MPI-EVA excavation the relevant layers include Layer 6-black (Upper Palaeolithic), Layer 7 (low number of artefacts; all from the contact zone with Layer 8, likely originating from Layer 8), Layers 8 and 9 (LRJ), Layers 10–12 (undiagnostic, potentially Middle Palaeolithic) and Layers 13–14 (archaeologically sterile limestone detritus). Notably Layer 6-black is clearly equivalent with the black Layer VIII, while Layers 8 and 9 represent the LRJ occupations corresponding to Hülle's grey Layer X, with the main LRJ occupation in Layer 8. In the TLDA/MPI-EVA excavation, a large rock of 1.7 m depth originating from a roof collapse event separated Layer 7 and 6 and largely displaced the equivalent of Hülle's middle brown Layer IX. Several large rocks from such roof collapse events restricted the size of the excavation in the lower layers (7 and below) to 1 complete, 2 half and 1 quarter squares, down from 18 squares at the surface of the 8 m deep sequence. However, the rockfall event sealing Layer 7 also provides ideal preservation conditions of the lower sequence in the area of the TLDA/MPI-EVA excavation, as the lower depositions were effectively protected from post-depositional disturbance.

Based on the newly produced radiocarbon chronology of the lower sequence of the site, the LRJ Layers 9 and 8 now date to 47,500–45,820 cal BP and 46,820–43,260 cal BP, respectively (modelled age ranges, 95% probability)[5]. The overlying Layer 7 dates to 45,890–39,110 cal BP while at the

base of the archaeological sequence Layer 11 has an age range of 55,860–48,710 cal BP[5].

Newly discovered human remains now link the LRJ at Ranis to *Homo sapiens* as the maker of this technocomplex[5]. A total of 13 *H. sapiens* skeletal fragments were identified through proteomic screening and in some cases through morphological analysis of previously unidentified small fragments and their taxonomic identity was confirmed using ancient DNA analysis. In the TLDA/MPI-EVA excavation three fragments were discovered in Layer 8 and one in Layer 9 (Figure 3). Additionally, nine fragments were recovered from the Hülle collection in material attributed to Layers IX, X, and XI (Figure 3). Excavation documentation shows that despite some attributions to Layer IX and XI, the fragments were recovered on the same day or within a day of recovery of LRJ artefacts in the same squares and are, therefore, most likely associated with the LRJ and were misassigned due to the excavation methodology[5]. This is confirmed by direct radiocarbon dates that were obtained for 6 of 9 *Homo sapiens* skeletal fragments from the Hülle collection, which all fall into the age range of Layers 8 and 9 of the TLDA/MPI-EVA excavation[5].

The faunal spectrum of the lower sequence, including the LRJ layers, is dominated by reindeer (*Rangifer tarandus*) and includes additional cold-adapted species such as wolverine (*Gulo gulo*), woolly mammoth (*Mammuthus primigenius*), woolly rhinoceros (*Coelondonta antiquitatis*) and arctic fox (*Alopex sp.*)[6]. Some species with a wide range of climatic flexibility including warmer environments, such as red deer (*Cervus elaphus*), are present, but in low numbers. In Layers 8 and 9 (LRJ), cave bear (*Ursus spelaeus*) remains are also abundant. Overall, the faunal composition indicates an accumulation in a cold steppe/tundra environment[6]. Evidence from zooarchaeological, palaeoproteomic and sediment aDNA analysis shows that human presence at the site fluctuated, with lowest human impact in Layer 7 and 10, while human modifications such as marrow fractures and cut marks are comparatively most abundant in the LRJ Layers 8 and 9, but still sparse (3.7–4.3 %)[6]. Overall, bone modifications from carnivores including gnaw marks and stomach

acid etching are dominant throughout the sequence[6]. Low numbers of hyena skeletal remains combined with high proportions of hyena (*Crocuta spelaea*) aDNA in sediments and abundant coprolite fragments suggest a use of the site as a hyena den for at least part of the site formation[6]. Human occupations of the site during the LRJ, but also the remaining lower sequence, appear to have been of low intensity either by small groups or through short or infrequent visits[6]. The relatively low density of stone tools in the LRJ layers supports this hypothesis[5].

2 Oxygen stable isotope palaeoclimatology

2.1 Oxygen palaeoclimatology background

The use of oxygen stable isotope measurements ($\delta^{18}O$) of tooth enamel of terrestrial mammals as a palaeoclimatic proxy is based on two fundamental principles. 1) There exists a strong linear relationship between air temperature and $\delta^{18}O$ in precipitation ($\delta^{18}O_{precip}$) and rain-fed water sources in mid- to high latitudes[7–12], where low $\delta^{18}O$ values correspond to low temperatures and high $\delta^{18}O$ values represent higher temperatures. 2) Animals that regularly drink large amounts of surface water from precipitation-fed water sources (such as aurochs/bison, horses, or mammoths) record the oxygen isotope composition of drinking water in the oxygen isotope composition of their tooth enamel[13–16]. In conjunction, these two relationships enable the reconstruction of palaeotemperatures using $\delta^{18}O$ values of faunal skeletal remains.

The isotopic composition of surface waters that are available for drinking is determined by multiple interwoven effects, but the oxygen isotope composition of precipitation ($\delta^{18}O_{precip}$) at mid- to high latitude locations generally shows a strong dependence on air temperature and this characteristic is inherited by precipitation-fed surface waters[7–12,17]. This temperature effect fundamentally establishes the role of $\delta^{18}O_{enamel}$ as a proxy for palaeotemperatures at mid- to high latitude

sites, where higher δ^{18}O in tooth enamel indicates higher air temperatures and low δ^{18}O values indicate lower temperatures. This holds true for geological time-scales of global climatic change as well as across annual seasonal cycles[9,10]. Oxygen isotope variation in precipitation and derived surface waters has been shown to be well-reflected in homeothermic mammals regularly consuming large amounts of liquid water, as drinking water δ^{18}O ($\delta^{18}O_{dw}$) is the most dominant driver of body water, and therefore, enamel δ^{18}O variability in such animals[13–16]. The specific linear relationship between enamel δ^{18}O and drinking water δ^{18}O is species-specific due to the influence of factors such as metabolic rate and body temperature[13].

Not only is $\delta^{18}O_{enamel}$ of such obligate drinking animals tied to $\delta^{18}O_{precip}$ and temperature conditions generally, δ^{18}O measurements of serially-sampled enamel can even be used to obtain sub-annually resolved and time-dependent information. Temperature-driven seasonal δ^{18}O fluctuations in precipitation are recorded as a sinusoidal pattern of δ^{18}O in sequential tooth enamel samples of animal teeth, with peaks indicating the summer season and troughs the winter season[18,19]. Extracting such time-dependent δ^{18}O patterns is possible due to the incremental formation of tooth enamel from the crown to the root-enamel-junction, after which the tissue does not remodel[20,21]. The stable isotope time series that can be obtained from a single tooth, therefore, covers the time of tooth enamel formation, which is usually 1.5 to >2 years in equids, depending on the tooth type and extent of tooth wear[18,22]. In this study, we measure δ^{18}O of sequentially sampled tooth enamel using the phosphate moiety of bioapatite ($\delta^{18}O_{phos}$), which is more resistant to diagenetic change than the carbonate fraction[23] and allows higher precision of palaeotemperature estimation as most modern calibration data has been measured using bioapatite phosphate[24].

In addition to comparing oxygen isotope measurements with existing data to make inferences about palaeotemperature conditions, we also use these measurements to quantitatively esti-

mate past temperatures. Oxygen isotope values can be compared directly with measurements generated in a comparable manner on remains of the same species to draw inferences on relative differences in seasonality (i.e. summer to winter differences) or temperature (warmer vs colder, more seasonal vs less seasonal). Such direct comparisons introduce the least amount of uncertainty, particularly if sampling approach and tooth types are also consistent. However, few δ^{18}O data from modern contexts or from Pleistocene sites are available for such comparisons, making this approach often insufficient for characterising past climates and contextualising obtained data.

Thus, a conversion to palaeotemperature estimates is almost always necessary to enable comparison with other species or data from other palaeoclimatic proxies, despite the known uncertainties that are involved. Palaeotemperature conversions are derived using formalised versions of the linear relationships between temperature and $\delta^{18}O_{precip}$, as well as between $\delta^{18}O_{dw}$ and $\delta^{18}O_{enamel}$ and employ error propagation methods to determine estimation uncertainty[14,15,24–29]. Regression equations of these linear relationships are established using modern calibration data from animals and drinking water as well as $\delta^{18}O_{precip}$ and air temperature measurements from measurement stations (see Supplementary Text 5). These relationships are well documented in modern calibration data, but their application to archaeological settings to reconstruct past climates relies on a number of assumptions that we discuss briefly here. Firstly, the exact numerical relationship between environmental water δ^{18}O and air temperature needs to be appropriately similar between modern and archaeological settings. This is to a certain degree dependent on the time stability of atmospheric circulation systems due to the role of moisture transport history. A sufficient degree of circulation similarity, and therefore stability of the slope of the $\delta^{18}O_{precip}$ - air temperature relationship, between modern-day and the Late Pleistocene in Europe has been shown using circulation models as well as by comparisons of palaeotemperature estimates with

$\delta^{18}O$ measurements of Pleistocene groundwaters[25,28,30–32]. Additionally, geospatial patterns in $\delta^{18}O_{enamel}$ in Europe in the Late Pleistocene reproduce current spatial patterns of west-east decrease in $\delta^{18}O_{precip}$ due to progressive rainout across the European continent from a westerly moisture source, further supporting a similarity in broad atmospheric circulation patterns[33]. Additionally, the $\delta^{18}O$ value of seawater influences $\delta^{18}O_{precip}$ and is known to change through time due to changes in global ice-volume[34]. During MIS 3 $\delta^{18}O$ of seawater was ~0.5 ‰ higher than it is today[34], which is a very small change in relation to the $\delta^{18}O$ difference between the values measured in this study and modern-day $\delta^{18}O$ values (in fauna or precipitation). At most, this effect would lead to a slight temperature over-estimation, and we argue that it is negligible in regard to this study.

Secondly, the relationship between $\delta^{18}O_{enamel}$ and $\delta^{18}O_{dw}$ needs to be stable over time to justify the use in palaeotemperature estimations. This relationship could change over time if 1) drinking requirements/behaviour changed dramatically, e.g. if an obligate-drinking species became non-water dependent, or 2) if metabolic characteristics such as metabolic rate or body temperature changed over time. There is no evidence to suggest that pronounced metabolic changes with the potential to impact species-specific offsets between $\delta^{18}O_{enamel}$ and $\delta^{18}O_{dw}$ or change drinking requirements have taken place in equids across the relatively short time scales since the Late Pleistocene[35–42]. We therefore consider that this assumption is valid. To further validate that study species $\delta^{18}O_{dw}$ reflects past $\delta^{18}O_{precip}$, and therefore climatic impacts, some studies recommend the use of multiple species for this analysis to check if any influence of drinking behaviour impacts $\delta^{18}O$ and to mitigate the uncertainties involved in estimating $\delta^{18}O_{dw}$ from $\delta^{18}O_{enamel}$[e.g. 24,43,44]. Unfortunately this was not possible in this study due to limitations of the available faunal material. Other than equids, the Ranis faunal collection contains almost no herbivore macrommal remains from species with large drinking requirements and teeth suitable

for sequential sampling. Suitable wolly mammoth, and large bovid (aurochs/bison) teeth are present in the collection, but such low numbers (< 5 in the whole collection) that analysis of these specimens would not yield meaningful information. Woolly rhinoceros teeth are present, but due to lack of information on tooth minerialization of this taxon it is not suitable for sequential sampling and would introduce unknown amounts of seasonal bias even into bulk samples. Similarly, Cervid teeth are more abundant but Cervid teeth do not record full annual cycles in their teeth and are therefore unsuitable for seasonal climatic reconstructions and can introduce seasonal bias into bulk samples unless pairs of second and third molars articulated in mandibles are available[45], which is not the case for the Ranis collection. However, $\delta^{18}O_{dw}$ values of equids have been shown to be in good agreement with those from a range of other taxa[43,46–48] and we have investigated in detail whether diet or drinking behaviour could have influenced equid $\delta^{18}O$ in section 2.2.

Other palaeotemperature estimation prerequisites should be validated for each study area. This includes the assumption that drinking water sources used by the analysed animals are predominantly fed by local precipitation and are isotopically closely tied to it. The role of aridity (leading to evaporation) in affecting $\delta^{18}O$ of potential water sources should also be checked for each study setting. These are discussed further in the following section.

2.2 Hydrotopographical setting and non-temperature effects on oxygen stable isotopes

For each study setting it needs to be established that $\delta^{18}O$ values of surface water sources in the region sufficiently reflect $\delta^{18}O_{precip}$, as precipitation measurements are used in the modern calibration data sets employed during palaeotemperature estimation. Indeed, the majority of open water source types in any given area normally relatively closely reflect $\delta^{18}O_{precip}$[17]. However, a

few specific water source types can isotopically deviate, sometimes substantially, from meteoric water. This particularly concerns deep groundwaters, large rivers or lakes, and glacial/snow melt due to effects of water transport, water residence time, evaporation, and selective incorporation of cold climate/winter season and/or high altitude precipitation[49–51]. Substantial water consumption from highly evaporated lakes would cause animals to exhibit high $\delta^{18}O_{enamel}$ values, which is in contrast to the uncommonly low oxygen isotope values of the animals from Ranis measured in this study. Combined with the absence of large lakes in the vicinity of the site or evidence of such from the Late Pleistocene, substantial consumption of evaporated lake water is unlikely. This leaves us to consider if large rivers, deep groundwater or glacial melt water are relevant.

Uncommonly low $\delta^{18}O$ values could be caused by consumption of glacial melt water or perennial snow, water transported from high latitudes or high altitudes, or through consumption of deep groundwater formed during colder climates. During MIS 3, or even MIS 4, both the Fennoscandian Ice Sheet and the Alpine glaciers were at least several hundred kilometres distant from Ranis and transport of glacial melt to the region by rivers is blocked by the regional mountain ranges[52]. Water transport from high elevations is similarly unlikely. The river most likely to transport higher elevation waters to the site vicinity is the Saale River, with a maximum elevation of 856 m in its catchment[53]. With an elevation difference of ~500 m compared to the site location, the water transported by the Saaler River would only be maximum ~0.5 ‰ higher than local $\delta^{18}O_{precip}$, and $\delta^{18}O$ measurements from the lower Saale catchment show it to be isotopically relatively similar to local $\delta^{18}O_{precip}$ (Extended Data Figure 3). Generally, the mountain ranges surrounding the site do not exceed elevations of 1000 m a.s.l. so a strong impact from high-elevation precipitation on locally available water sources is unlikely. Finally, a consumption of deep groundwater is possible. We argue that this is also unlikely, as such groundwaters rarely feed surface springs as these aquifers are normally relatively far below the surface. Additionally, such water sources

are by definition heavily time-averaged and show essentially no seasonal variability. Regular consumption of groundwaters would therefore cause an absence of a seasonal sinusoidal δ^{18}O signal in tooth enamel, which we do not observe in the equids studied here. A similar case can be made for potential consumption of perennial snow. While this could well have existed in the landscape around the site, snow consumption should only substantially distort seasonal δ^{18}O signals if consumed outside the winter season. However, this should logically also produce a noticeably damped seasonal δ^{18}O that is incompatible with the observed data. Overall, we therefore argue the available water sources around Ranis should reflect $\delta^{18}O_{precip}$ reasonably well.

Finally, it should be considered whether δ^{18}O changes over time that we observe in our data could be driven by non-temperature effects on $\delta^{18}O_{precip}$. As we have already considered circulation-related effects, aridity/rainfall amount remain as a final potentially substantial driver of δ^{18}O. Changes in δ^{15}N throughout the Ranis sequence suggest that aridity or changes in soil water availability could play a role, but negative correlation between δ^{15}N and δ^{18}O demonstrate that δ^{18}O values are not affected by this, as high aridity should produce high δ^{18}O during high δ^{15}N phases. At the same time, δ^{18}O values in this study are remarkably low, which is more consistent with low temperature and low-evaporation environments. If evaporative enrichment played a substantial role in influencing δ^{18}O, this would mean that temperatures were even lower than reconstructed. As evaporative enrichment even at the reconstructed temperatures would have been extremely limited, it seems unlikely that even lower temperatures could have been accompanied by sufficient evaporative enrichment to elevate δ^{18}O through aridity effects and cause a temperature over-estimation. Conversely, lowering of δ^{18}O through high rainfall amounts is also inconsistent with the high δ^{15}N values during times of particularly low δ^{18}O, as well as with the vast majority of palaeoclimatic reconstructions from other proxies all indicating much drier conditions for MIS 3 stadials[54,55].

At the same time, the decline in total organic carbon and total nitrogen content of sediments from Layer 9 to 7 of the Ranis sequence supports that the co-occurring drop in δ^{18}O that we observe is driven by a temperature decline[5] (see main text for discussion). Additionally, particularly this δ^{18}O decline is congruent with climate cooling associated with Greenland Stadial 12 documented in pollen sequences, geochemical records, and earthworm calcite δ^{18}O records from west and southwest Germany, further supporting an interpretation of our δ^{18}O data in relation to temperature change[54–57]. Therefore, an interpretation as a palaeotemperature proxy is most logical.

3 Strontium isotopes and animal spatial ecology

The strontium isotope ratio (^{87}Sr/^{86}Sr) in tooth enamel of terrestrial animals ultimately reflects ^{87}Sr/^{86}Sr of bioavailable Sr from underlying bedrock and derived soil[58]. The ^{87}Sr/^{86}Sr ratios of different lithologies are in turn driven by bedrock age and the ^{87}Rb content at the time of formation[59]. This is because ^{87}Sr is a radiogenic isotope that is produced through the decay of ^{87}Rb over time[59]. This mechanism means that older bedrocks typically tend to exhibit higher ^{87}Sr/^{86}Sr than younger rocks (of similar lithology). Geogenic ^{87}Sr/^{86}Sr of bedrock is subsequently transferred to soils through bedrock erosion/weathering and bioavailable strontium is taken up from the soil by plants and transferred through the food web from herbivores to omnivores and carnivores[60,61]. Isotopic fractionation of Sr from biological processes is negligible and, consequently, ^{87}Sr/^{86}Sr effectively does not change from plants to herbivores or further up the trophic chain[61,62]. Thus, ^{87}Sr/^{86}Sr in tooth enamel represents a record of the lithologies that animals range over and can be used to trace animal movements and characterise their spatial ecology[58]. Mobility or place-of-origin information can be extracted either from bulk samples of enamel, representing the average ^{87}Sr/^{86}Sr composition incorporated throughout enamel formation,

or from sequential samples to extract a time-dependent $^{87}Sr/^{86}Sr$ measurement series[63]. As animal migrations most commonly occur seasonally with movements between summer and winter range locations, a comparison between a summer and a winter sample can be used as a check for long-distance or altitudinal migrations[64]. This approach offers lower resolution than full sequential $^{87}Sr/^{86}Sr$ profiles but is useful as a control to detect any migration-induced biases of $\delta^{18}O$. As geospatial patterns in $\delta^{18}O$ are characterised by low rate-of-change gradients over large distances or altitudinal gradients, this approach is normally sufficient to detect migratory behaviours that would be relevant from an oxygen isotope perspective.

We use this summer/winter comparison approach on sequentially sampled equid specimens to confirm that $\delta^{18}O$ values are representative of local conditions without bias from long-distance migrations. For other fauna, bulk samples were analysed for $^{87}Sr/^{86}Sr$ to evaluate the relationship between $^{87}Sr/^{86}Sr$ and $\delta^{66}Zn$ and establish any potential influence of bedrock lithology on $\delta^{66}Zn$ in our study setting (see Supplementary Text 4). The $^{87}Sr/^{86}Sr$ ratios of equids in our study range from 0.7090 to 0.7120. Most specimens fall into the higher end of this value range, between ~ 0.710–0.712 (Supplementary Figure 3). These values match $^{87}Sr/^{86}Sr$ ratios reported for lithologies in Thuringia. A study of bioavailable strontium in northern Thuringia reported $^{87}Sr/^{86}Sr$ ratios in tree leaves ranging from 0.70817 in Triassic carbonates (Muschelkalk) to 0.71116 in Triassic sandstones (Buntsandstein)[65]. South-eastern Thuringia also contains several granitic geological units with high $^{87}Sr/^{86}Sr$ values up to ~ 0.730, some of which are located within 50 km of Ranis[66], and this variability is also shown on a predicted strontium isoscape (Supplementary Figure 4). A study of surface water samples further reported $^{87}Sr/^{86}Sr$ ranging from 0.7091 to 0.71381 at different points along the upper course of the Saale River through the Thuringian-Franconian Slate Belt[67], where this river passes before running close to the site of Ranis. The $^{87}Sr/^{86}Sr$ value range of equids is additionally fully encompassed by values observed in hyenas and ursids (0.7083 to

0.7133) – taxa that have documented small to modest range sizes in Late Pleistocene and modern case studies, particularly in the case of cave bears[68–70] (Supplementary Figure 3). The $^{87}Sr/^{86}Sr$ of equids is therefore completely encompassed by measured $^{87}Sr/^{86}Sr$ of plants and water samples, predicted bioavailable $^{87}Sr/^{86}Sr$ and $^{87}Sr/^{86}Sr$ of animals with likely small home range. This means that equid $^{87}Sr/^{86}Sr$ are consistent with a non-migratory ranging behaviour and a presumable habitat within a few tens of kilometres of Ranis. The relatively large range of $^{87}Sr/^{86}Sr$ values represented within the complex geology of Thuringia and in our equid samples means that there likely is significant overlap with lithologies further away from the site and comparisons of $^{87}Sr/^{86}Sr$ values between animals and lithologies cannot fully exclude a non-local origin. However, to our knowledge no spatial ecology study of Eurasian Late Pleistocene equids has so far found any evidence for seasonal long-distance migrations[71–73]. Furthermore, summer and winter $^{87}Sr/^{86}Sr$ of the Ranis equids are very similar with seasonal $^{87}Sr/^{86}Sr$ differences < 0.0005 in almost all cases (Supplementary Figure 3) indicating that summer and winter ranges were located on similar lithologies. Therefore, a non-migratory spatial ecology is the most parsimonious for these equid specimens. One exception, specimen R10131, shows a larger seasonal difference in $^{87}Sr/^{86}Sr$, accompanied by an unusually larger seasonal difference in $\delta^{66}Zn$, which could also be affected by bedrock lithology to some extent (Supplementary Figure 6; Supplementary Text 3). The $^{87}Sr/^{86}Sr$ ratios of this individual still fall within the range of values observed in other equids and predators with typicall small home ranges as well as eastern Thuringian bedrock types (Supplementary Figure 3). We therefore argue that long-distance migration is still fairly unlikely. However, due to the larger seasonal $^{87}Sr/^{86}Sr$ change, we cannot fully exclude a more migratory ecology and thus exclude this specimen from climatic interpretations of $\delta^{18}O$ and dietary interpretations of $\delta^{66}Zn$.

4 Zinc stable isotopes

4.1 Principles of zinc stable isotope variability

Zinc stable isotope analysis falls into the group of non-traditional stable isotope analyses and has been established fairly recently as a tracer of diet and ecology of terrestrial and marine vertebrates[74–81]. Zinc is a transition metal but due to it's full d-shell (10 electrons in the d-shell), it is in many respects chemically similar to alkaline earth metals such as magnesium. Zn has five naturally occurring stable isotopes and measurements of several different isotope ratios have been conducted with ^{66}Zn/^{64}Zn, expressed as δ^{66}Zn, being the most commonly used and most established.

Zinc is an essential trace element occurring in low abundances, but it serves important biological functions where it forms metal organic compounds that are involved in a number of enzymes and regulatory proteins[82]. Indeed, zinc availability in soils is one of the most important limiting factors for plant growth due to its essential role in cell wall formation[83]. Zinc stable isotope variability in terrestrial food webs reflects both baseline variability in soils and plants as the ultimate source of zinc introduced to the animal biome, as well as biological fractionation within the food web[84–87]. In soils, δ^{66}Zn variability mainly is driven by the lithology of the underlying bedrock, with igneous rocks exhibiting very uniform δ^{66}Zn values of 0.3 $\pm$ 0.14 ‰, while sedimentary rocks show a wider range of variability from ~-0.5 to 1.5 ‰ depending on their formation history[88,89]. Highest δ^{66}Zn values of up to 1.4 ‰ occur in marine carbonates, as these biogenic lithologies are formed from calcifying marine organisms whose tissues exhibit higher δ^{66}Zn values due to biological isotopic fractionation[90]. Additional zinc isotope variability in soils may be introduced by the source of organic matter in the soil as well as zinc biogeochemical cycling[87,88]. Plants take up zinc from the soil, with initial fractionation from soil to roots leading

to higher δ^{66}Zn values in roots[84–86]. Further fractionation occurs in plants during zinc transport, as cells appear to preferentially take up the heavier Zn isotopes while the lighter isotopologues are preferentially transported to the more aerial or peripheral parts of the plant[84–86]. This results in an isotopic differentiation between plant parts as well as between different plant types. Within a single plant upper plant parts such as tree leaves exhibit lower δ^{66}Zn values compared to stems and across plant species higher growing species such as shrubs or trees show lower δ^{66}Zn values compared to higher δ^{66}Zn in lower growing species such as grasses or herbaceous plants[84–86]. Zinc isotope variability in the plant biome is subsequently passed on to herbivorous consumers and in turn from herbivores to predatory animals with further isotope fractionation in these organisms[75,76,81].

In animals, diverging δ^{66}Zn values are observed between different tissue types with soft tissues, such as muscle or kidney, showing lower δ^{66}Zn and hard tissues, such as bone, exhibit higher δ^{66}Zn[81,91]. This effect is the basis of a trophic level effect in δ^{66}Zn, where δ^{66}Zn decreases along the trophic chain as predators consume soft tissues of prey organisms[75,79,81]. In this context it needs to be kept in mind that bone generally exhibits higher δ^{66}Zn than soft tissue, which shifts δ^{66}Zn of bone-eating carnivores such as Hyaenidae towards higher δ^{66}Zn values normally observed in omnivores[75,76,91]. Additionally, nursing and *in utero* formation has been shown to impact tooth enamel δ^{66}Zn values, where δ^{66}Zn values of teeth formed during nursing or *in utero* are higher than those formed post weaning[92]. This is often relevant especially for carnivora δ^{66}Zn values, as these animals tend to form many of their teeth during the nursing period, and timing of enamel formation is often not well studied.

Several studies have investigated the ability of tooth enamel and enameloid bioapatite to preserve biogenic δ^{66}Zn values over archaeological and geological time scales[76,78,80,93]. These studies have demonstrated that δ^{66}Zn is preserved and that neither Zn substantial uptake nor leach-

ing occurs in these tissues including in tropical conditions and for up to tens of millions of years, into the Miocene[76,80]. This has been achieved by evidencing the preservation of biogenic Zn concentration spatial profiles, relationship with other trace element concentration profiles, positive correlation with δ^{15}N data, a lack of relationship between isotopic and concentration data, comparisons with dentine samples, and reliable reproduction of known trophic level relationships and trophic level spacings in past food webs[76,80,93]. As recommended in ref.[76] we use the relationship between zinc concentration and isotopic data as a diagenetic check, as this relationship should evidence a mixing line if enamel bioapatite has been subject to zinc leaching or uptake of exogenous zinc from the burial environment.

4.2 Impacts of bedrock lithology on zinc stable isotopes in Ranis

Studies of δ^{66}Zn in different food webs generally indicate that diet is by far the dominant driver of δ^{66}Zn. Nonetheless as a more minor effect, small baseline differences in δ^{66}Zn have been noted between some food webs related to differences in bedrock lithology[77]. However, baseline differences generally appear to be insignificant if sites are located on similar geological units, as was shown in a comparison of two sites located ~150 km apart on karstic geology in Laos[77]. At these sites, an inverse correlation between δ^{66}Zn and ^{87}Sr/^{86}Sr across all faunal specimens was observed, driven by the presence of high δ^{66}Zn/low ^{87}Sr/^{86}Sr carbonate lithology and low δ^{66}Zn/high ^{87}Sr/^{86}Sr granitic lithologies in the study area[77]. In our data from Ranis, we observe a very weak inverse relationship between δ^{66}Zn and ^{87}Sr/^{86}Sr, which is statistically significant (as established by Pearson correlation), but appears mostly driven by two outliers and has a very small slow coefficient (Supplementary Figure 8). We therefore argue that δ^{66}Zn is predominantly driven by dietary groups in our data, with perhaps a very small influence related to ranging across different geological units including carbonate and granite lithologies present in the area (Sup-

plementary Figure 4, Supplementary Text 3). The range of $^{87}Sr/^{86}Sr$ observed in the food web is consistent with values reported for lithologies within a few tens of kilometres of Ranis, indicating that animals ranged over local landscapes and were unlikely to have undertaken long-distance migrations, precluding stronger non-dietary impacts on $\delta^{66}Zn$ (Supplementary Text 3). However, a single equid individual (R10131), exhibits an unusually large $^{87}Sr/^{86}Sr$ difference between summer and winter values (summer and winter samples determined using $\delta^{18}O$ data; Supplementary Figure 3). As discussed in Supplementary Text 3, while long-distance migration remains unlikely for this individual, the larger seasonal $^{87}Sr/^{86}Sr$ means that seasonal movement is a possibility for this specimen. Interestingly, this same specimen also exhibits by far the largest seasonal amplitude in $\delta^{66}Zn$ (Supplementary Figure 5, Supplementary Figure 6). Due to this co-occurrence of large seasonal amplitudes, we argue that movement across different geological types may have influenced seasonal $\delta^{66}Zn$ differences in this case, although further research into seasonal variation of $\delta^{66}Zn$ in herbivores is needed to further clarify this. If a movement across bedrock geologies influenced $\delta^{66}Zn$ in this individual, this means that it would have moved from a high $\delta^{66}Zn$ lithology in summer to a lower $\delta^{66}Zn$ lithology in winter, while $^{87}Sr/^{86}Sr$ values for both seasons are relatively high ($^{87}Sr/^{86}Sr_{summer}$ = 0.7112; $^{87}Sr/^{86}Sr_{winter}$ = 0.7120; Supplementary Figure 5, Supplementary Figure 3). High $^{87}Sr/^{86}Sr$ values mean that a carbonate bedrock is very unlikely to be the cause of high $\delta^{66}Zn$, as marine carbonates exhibit $^{87}Sr/^{86}Sr$ between ~0.707 and 0.709[94]. Conversely, granitic geologies can be excluded due to their uniformly low $\delta^{66}Zn$. No $\delta^{66}Zn$ values are available for the lithologies around Ranis and data generally is sparse, making it difficult to determine what type of geology would match high $\delta^{66}Zn$ and high $^{87}Sr/^{86}Sr$. As $\delta^{66}Zn$ has been reported to be very variable for sedimentary rocks, a high $^{87}Sr/^{86}Sr$ sandstone may for instance be a candidate. Alternatively, it is possible that such isotopic characteristics could match some of the shales found in Thuringia and Saxony, as high $^{87}Sr/^{86}Sr$ has been reported for some of these fine-grained siliclastic rocks and high $\delta^{66}Zn$ have been reported for black shales elsewhere[95,96].

More specific δ^{66}Zn data for sedimentary rocks from Germany would be needed to support these hypotheses. As such candidate geologies are still present within ~100 km of Ranis, we maintain that long distance migration of this individual remains fairly unlikely. However, as δ^{66}Zn could be influenced to some degree by movements across different bedrock types and thus potentially be biased relative to a purely dietary signal, we have chosen to exclude the R10131 specimen from interpretations of δ^{66}Zn in relation to dietary ecology. Similarly, we have not considered this individual for palaeoclimatic reconstructions using δ^{18}O, to avoid any bias related to geospatial variation of δ^{18}O.

4.3 Zinc stable isotopes and herbivore feeding ecology

Trophic level inference has so far been the focus of zinc stable isotope applications in the few archaeological and palaeontological applications that have been developed very recently[76,77,80,93]. However, it is becoming increasingly clear that substantial isotopic variability exists within herbivores as well. In particular, some of the published zinc stable isotope data of terrestrial herbivores commonly show comparatively low δ^{66}Zn values in taxa associated with closed habitats and a stronger browse component in the diet, such as cervids[75,76,93]. Higher δ^{66}Zn values on the other hand have been observed in equids, which are more commonly associated with grazing and open environment feeding[75,93]. This pattern is also observed in the food web from Ranis studied here (Extended Data Figure 2). This may suggest that herbivores reflect the δ^{66}Zn patterning in plants where tree leaves show lower δ^{66}Zn than grasses or herbaceous plants[84–86]. This would in turn also indicate that δ^{66}Zn diachronic changes within a single herbivore species over time may reflect dietary shifts along a grazer-browser spectrum. However, not all case studies conducted so far demonstrate a clear grazer/browser distinction in δ^{66}Zn. While a grazer/browser difference in δ^{66}Zn was clearly present in a modern eastern African food web[75] and a Late Pleistocene

food web in Spain[93], for example, data from Late Pleistocene sites in Laos did not show a clear δ^{66}Zn separation of grazers and browser across a large number of herbivore taxa[76,77]. In these case studies from Laos, dietary group was initially inferred via δ^{13}C, but the expected δ^{66}Zn pattern was only observed between certain grazers and browsers, while additional δ^{66}Zn clustering within browsers was shown[76]. At this time, it remains unclear why grazer-browser patterning is observed in some settings but not others. The δ^{66}Zn groupings within herbivores do not seem to be related to digestive physiology (ruminants vs non-ruminants)[76,77], and the Ranis data further confirm this with non-ruminants falling both on the low (woolly rhinoceros) and high end (equids) of the herbivore δ^{66}Zn range (Extended Data Figure 2). Similarly, δ^{66}Zn variability in herbivores appears to be unrelated to body mass[76,77]. A lack of true specialist grazers at the sites in Laos and a higher proportion of C3 (rather than C4) grasses in the landscape may obfuscate grazer/browser δ^{66}Zn patterns in those case studies if grazer/browser distinctions are initially based on δ^{13}C, but more well-controlled modern data or cross-comparisons with other data on grazing/browsing behaviour are needed to definitively clarify the drivers of δ^{66}Zn in herbivores.

In the Ranis data high δ^{66}Zn values in equids compared to traditionally browser/mixed feeder cervid taxa are consistent with a grazer/browser pattern in δ^{66}Zn. At the same time, the relationship between equid δ^{66}Zn and environmental and climatic inferences from the other stable isotope tracers could further support that equid δ^{66}Zn is consistent with such a pattern. In equid samples, highest δ^{66}Zn co-occur with lowest δ^{18}O values and highest δ^{15}N values at ~43–45 ka cal BP (Figure 1, excluding R10131 due to potential seasonal movement; see discussion of spatial ecology above). Exceptionally low δ^{18}O values combined with high δ^{15}N values during this time suggest cold temperature conditions and a grazing specialisation in equids or potentially the presence of dry soils (see main text). Combined with δ^{13}C values characteristic of an open environment the three stable isotope tracers all suggest an open grassland environment, matching

data from pollen sequences from western and southwestern Germany[54–56]. An interpretation of high δ^{66}Zn values as being indicative of a grazer feeding ecology would thus fit well with the rest of the stable isotope data. Additionally, we observe a statistically significant correlation between δ^{66}Zn and δ^{15}N in equids (Supplementary Figure 12). Herbivore δ^{15}N in modern periarctic or past glacial/stadial ecosystems is commonly interpreted as either varying with grazer/browser diet or with bacterial activity in the soil, in turn driven by water availability, temperature and presence/absence of permafrost, and we argue here that a relationship with grazing specialisation or with soil water availability most likely explains the patterns in our data (see main text). A positive correlation of δ^{66}Zn and δ^{15}N would therefore also be consistent with a grazer/browser patterning in δ^{66}Zn. If true, this would in turn mean that the degree of summer/winter δ^{66}Zn difference would be linked to seasonal dietary changes. In our data set we don't observe a fixed seasonal δ^{66}Zn pattern, and summer δ^{66}Zn values are higher than winter in some cases, but lower in others (Supplementary Figure 5). However, we do observe that individuals with higher δ^{66}Zn values are more likely to exhibit very small summer/winter δ^{66}Zn differences. This could perhaps indicate that equids with a grazer feeding ecology were grazing year-round with a strong degree of specialisation, while more mixed-feeding equids showed a higher degree of seasonal dietary flexibility.

However, at this point a grazer/browser interpretation of δ^{66}Zn is not sufficiently demonstrated, as both tracers are also influenced by biogeochemical cycling in the soil (see main text and background discussion above). The precise environmental and climatic impacts on δ^{66}Zn variability in soils are poorly understood[84,87], but the known effects of biological processes and nutrient cycling on soil δ^{66}Zn mean that such impacts could potentially lead to a positive correlation of δ^{66}Zn and δ^{15}N in soils and plants.

Overall, we therefore conclude that our herbivore δ^{66}Zn data are tentatively consistent with a

grazer/browser patterning in δ^{66}Zn, but other explanatory mechanisms related to processes in the soil may play a role as well. Our data are not sufficient on their own to demonstrate or falsify the validity of either approach but may be useful as a foundation for further ground-truthing.

5 Extended methods

5.1 Tooth enamel sampling

5.1.1 Sequential powder sampling

Prior to enamel sampling, dental calculus was removed from the sampling surface by abrasion with a tungsten carbide burr and using air abrasion with Korox 99,6% aluminium oxide (50 µm mesh size). After mechanical cleaning, all teeth were cleaned in Milli-Q ultrapure water and left to dry before sampling. Tooth enamel samples were drilled with a diamond tipped dental drill bit as small strips perpendicular to the tooth growth axis spaced ~3mm apart in a series covering the complete tooth crown. Sample strips commonly had dimensions of 1 mm in cervico-occlusal length and 5 mm mesio-distal width and a depth between 0.5 and 1 mm, usually encompassing ~80% of the total enamel thickness. Drill bits were cleaned between samples using 1 M nitric acid and two 5-minute ultrasonication cycles in Milli-Q ultrapure water. Sampling was conducted in a positive pressure laminar flow box to avoid contamination and, thus, enable use of powder samples for strontium and zinc stable isotope analysis, in addition to oxygen stable isotope analysis.

5.1.2 Bulk piece sampling

When bulk sampling of an enamel piece was undertaken, the surface was first mechanically cleaned using a handheld drill equipped with a diamond-tipped burr to remove any adhering external material. Subsequently, enamel samples for each specimen were collected at the base of the crown using a diamond-tipped cutting wheel. The amount of material collected varied slightly due to differences in the enamel's relative thickness and the tooth's overall aspect but was typically around 10 mg. Dentine attached to the inner portion of the enamel was then mechanically removed using a handheld drill equipped with a diamond-tipped burr. As with sequential powder sampling, sampling was conducted in a positive pressure laminar flow box, and the cutting wheels and burrs were similarly cleaned between samples.

5.2 Oxygen stable isotope analysis

5.2.1 Silver phosphate preparation

Approximately 5 mg of tooth enamel powder of each sample were weighed into 2 mL Eppendorf microcentrifuge tubes. Samples were agitated in 0.4 mL of 2 M hydrofluoric acid (HF) for 24 hours to digest bioapatite and remove calcium from the solution as CaF_2. After digestion, samples were centrifuged (12000 rpm for 5 Min) to separate the phosphate containing solution from CaF_2 precipitate and the supernatant solution was then transferred to a clean microcentrifuge tube. To maximise phosphate recovery the CaF_2 precipitate was washed once with 0.1 mL MilliQ ultrapure water, and the wash added to the phosphate-containing solution. The sample solution was then titrated to neutrality as indicated by the colour change point of Bromothymol Blue indicator using 25% ammonia solution (NH_4OH). 60 μL of NH_4OH were first added to each sample using an automatic pipette and each solution then slowly brought to the colour change point (yellow to

green) using individual drops of NH_4OH added with a 100 μL Hamilton Microliter fixed needle syringe (Hamilton Bonaduz AG, Switzerland). From the neutralised solutions, Ag_3PO_4 was rapidly precipitated by addition of 0.4 mL 2 M silver nitrate ($AgNO_3$) solution. The resulting precipitate was separated from the supernatant solution by centrifugation (12000 rpm for 5 Min) and the remaining silver nitrate solution removed. The silver phosphate precipitate was then washed four times with 1 mL Milli-Q ultrapure water using centrifugation and vortex mixing steps between rinses to wash out any remaining silver nitrate from the sample. Silver phosphate samples were then dried overnight at 50 °C and stored in an evacuated desiccator until further analysis.

5.2.2 Oxygen stable isotope measurements

Phosphate oxygen isotope measurements of Ag_3PO_4 were conducted using a high temperature elemental analyser (TC/EA) coupled to a Delta V isotope ratio mass spectrometer via a Conflo IV interface (Thermo Fisher Scientific, Bremen, Germany) at the Max-Planck-Institute for Evolutionary Anthropology (MPI-EVA). Approximately 0.5 mg of each silver phosphate sample was weighed into cleaned silver capsules (3x4 mm, IVA Analysentechnik, Meerbusch, Germany) and introduced to the TC/EA using a Costech Zero Blank Autosampler (Costech International, Cernusco sul Naviglio, Italy). Conversion to CO was achieved using a reactor temperature of 1450 °C and gases separated using an Eurovector E11521 1.4 m x 4 mm x 6 mm stainless steel GC column with 80/100 mesh 5 Å molecular sieve packing (Eurovector Instruments & Software, Pavia, Italy) maintained at 120 °C with a carrier gas pressure of 1.3 bar. Samples were measured in triplicate, but in rare cases individual measurements were rejected if they did not meet quality control criteria such as appropriate peak area to sample amount relationship. In such cases $\delta^{18}O$ values are only based on two replicates.

Oxygen isotope delta values were two-point scale normalised to the VSMOW scale using matrix-

matched standards calibrated to international reference materials and scale normalisation was checked using three separate quality control standards. Scale normalisation was conducted using the B2207 silver phosphate standard (δ^{18}O = 21.7 ± 0.3 ‰, 1 s.d.; Elemental Microanalysis, Okehampton, UK) and an in-house silver phosphate standard (KDHP.N, δ^{18}O = 4.2 ± 0.3 ‰, 1 s.d.). This in-house standard was produced in 2017 by equilibrating a KH_2PO_4 solution made with Leipzig winter precipitation at ca. 140 °C for several days, after which the solution was neutralised using a small amount of NH_4OH and the phosphate precipitated as silver phosphate by addition of $AgNO_3$ solution. An equivalent method is described in detail in ref.[97]. The accepted value of this in-house standard was determined by two-point calibration using B2207 and IAEA-SO-6 (barium sulphate, δ^{18}O = -11.35 ± 0.3 ‰, 1 s.d.).

Aliquots of an in-house modern cow enamel standard (BRWE.2) and the standard reference material NIST SRM 120c (formerly NBS 120c) were precipitated and measured alongside each batch of samples to ensure equal treatment. Additionally, a commercially available silver phosphate (AS337382, Sigma Aldrich, Munich, Germany) was used as a third quality control standard to check across-run consistency of scale normalisation independent of silver phosphate precipitation. Measurements of these standards gave δ^{18}O values of 14.5 ± 0.4 ‰ for BRWE.2 (1 s.d., n = 72), 21.5 ± 0.7 ‰ for NIST SRM 120c (1 s.d., n = 85) and 13.8 ± 0.2 ‰ for AS337382 (1 s.d., n = 264). This compares well to the consensus value for NIST SRM 120c of 21.7 ‰, as well as the long-term averages for BRWE.2 of 14.5 ± 0.3 ‰ and 13.9 ± 0.2 ‰ for AS337382. Samples were usually measured in triplicate and average reproducibility of sample replicate measurements was 0.25 ‰. Consecutive analysis of sets of standards with widely spaced isotopic values showed no detectable memory effect and consequently no memory effect correction was required. No effect of the blank, of the sample amount, or peak height on the results was observed and consequently no blank correction or linearity correction was applied. If drift across the course of a run was

detected in the quality control standard AS337382, a linear drift correction based on the drift of both normalisation standards was used and checked with the quality control standard.

5.3 Zinc and strontium stable isotope analysis

Zinc and strontium extraction were both conducted on ~10 mg of tooth enamel powder of samples that represent the seasonal summer and winter (determined by oxygen isotope measurements). A total of 56 samples were processed, a summer and winter sample from each of the 16 directly dated equid specimens (total of n = 32) and an additional 24 samples from mammalian herbivore, omnivore, and carnivore taxa (Supplementary Table 1). Samples were digested in 2 mL of 65% HNO_3 (Merck SupraPur grade, Merck, Darmstadt, Germany) for 2 hours at 120 °C and the solution then evaporated to dryness. The resulting residue was re-dissolved in 1 mL of 3N HNO_3 at 120 °C for 1 hour. The sample solution was then loaded onto a 0.5 cm bed of cleaned and pre-conditioned 50-100 μm Sr-spec resin (EiChrom Technologies, Lisle, USA) placed in cleaned microcolumns. All sample solutions were passed through the column step 4 times to ensure complete loading of strontium onto the ion-exchange resin. Matrix elution was achieved using 3 times 400 μL of 3N HNO_3, after which strontium was eluted from the columns using 1.5 mL of MilliQ ultrapure water. The resulting Sr containing solution was evaporated to dryness at 120 °C and re-dissolved in 1 mL of 3 % HNO_3. Two process blanks were prepared alongside samples to assess the level of contamination during the sample preparation procedure. Additionally, two aliquots of the NIST SRM 1486 bone meal standard were treated with the samples to serve as a quality control standard. All samples were analysed for $^{87}Sr/^{86}Sr$ using a Neptune Multi-Collector Inductively Coupled Plasma Mass Spectrometer (MC-ICPMS, Thermo Fisher Scientific, Bremen, Germany) at the MPI-EVA. $^{87}Sr/^{86}Sr$ measurements were normalised for instrumental mass bias to $^{88}Sr/^{86}Sr$ = 8.375209 (exponential law) and corrected for ^{87}Rb interference. External data nor-

malisation was conducted using the NIST SRM 987 reference material ($^{87}Sr/^{86}Sr$ accepted value = 0.710240[98]; average of measured values = 0.710283 $\pm$ 0.000011 (1 s.d., n = 56)). Measurements of NIST SRM 1486 gave an average value of 0.709300 $\pm$ 0.000019 (1 s.d., 20 measurements of 10 aliquots), which is very close to the expected value of 0.709299 $\pm$ 0.000027 (in-house long-term average). A subset of samples (n = 41) was measured in duplicate with an average reproducibility of 0.0000077 (1 s.d.). A lack of mixing line in a plot of 1/concentration and $^{87}Sr/^{86}Sr$ values indicates that samples did not take up substantial amounts of Sr from the burial environment and therefore are unlikely to be diagenetically altered (Supplementary Figure 15).

For Zn extraction and purification samples were digested in 1 mL of HCl 1.0 N, then evaporated and dissolved again in 1.5 M hydrobromic acid (HBr). Zn was then purified in a two-step ion exchange chromatography method. Briefly, Zn was purified in pre-conditioned microcolumns on 1 mL AG-1x8 resin (200-400 dry mesh size, 106-180 µm wet bead size) using 2 mL of 1.5 M HBr for matrix residue elution and 5 mL of 0.5 M HNO_3 for Zn elution. Samples were then dried and re-dissolved in 1 mL 0.5 M HNO_3. For quality control purposes, aliquots of in-house reference material NIST SRM 1486 bone powder and/or the NIST SRM 1400 bone ash as well as procedural blanks were processed with all sample batches. Zinc isotope ratios were measured using Cu doping on a Neptune Multi-Collector Inductively Coupled Plasma Mass Spectrometer (MC-ICPMS, Thermo Fisher Scientific, Bremen, Germany) at the MPI-EVA. A subset of samples was analysed in duplicate and with an average analytical precision of 0.008 ‰. To assess preservation, we evaluated if samples showed any relationship between zinc concentration and δ^{66}Zn. A mixing line is not observed, indicating that samples preserved a biogenic δ^{66}Zn signal without uptake of exogenous zinc (Supplementary Figure 14).

The reference material Zn Alfa Aesar solution was used for standard bracketing. All δ^{66}Zn values are expressed relative to the JMC-Lyon standard with a mass dependent Alfa Aesar offset of

+0.27 ‰ for δ^{66}Zn[75,99]. Zinc concentrations were estimated based on Zn signal intensity (V) of the respective sample in comparison to three solutions with known Zn concentrations. Bracketing standard reproducibility within a session was always 0.03 (2 s.d.) or better. Reference materials NIST SRM 1400 and NIST SRM 1486 prepared and analysed with the samples had δ^{66}Zn values of 0.96 ± 0.06‰ (n = 8) and 1.20 ± 0.01‰ (n = 4) as reported elsewhere[78,92]. All samples and references materials show a Zn mass-dependent isotopic fractionation, i.e., the absence of spectral interferences as δ^{66}Zn vs. δ^{67}Zn and δ^{66}Zn vs. δ^{68}Zn values fall onto lines with slopes close to the theoretic mass fractionation values of 1.5 and 2, respectively. Measurement data for δ^{67}Zn and δ^{68}Zn can be obtained from the electronic data files of this study, which can be found at https://osf.io/wunfd/.

5.4 Collagen extraction and radiocarbon dating

Mandible bone or tooth dentine pieces with weights ranging from ~300–600 mg were sampled from equid specimens using a diamond coated rotary disk, after mechanical cleaning using air abrasion with Korox 99,6% aluminium oxide (50 µm mesh size). Collagen extraction followed an acid-base-acid plus ultrafiltration protocol at the Department of Human Evolution at the MPI-EVA following the protocol in refs.[100,101]. Samples were demineralised in HCl 0.5 M at 4 °C until soft and CO_2 effervescence had stopped, with HCl changed twice per week. The demineralised samples were treated with NaOH 0.1 M for 30 min to remove humic acid contamination and then re-acidified in HCl 0.5 M. Samples were then gelatinised in HCl pH3 at 75 °C for 20 h before being filtered to remove particles >60–90 µm (Ezee filters, Elkay Labs, UK) and ultrafiltered to concentrate the >30 kDa fraction (Sartorius VivaSpin Turbo 15). Filters were precleaned prior to use[102]. The >30 kDa fraction was lyophilised for 48 h and the collagen was immediately weighed to determine the collagen yield as a percentage of the dry sample weight. The quality of result-

ing collagen extracts was assessed prior to radiocarbon dating using collagen yield, carbon and nitrogen elemental concentration, and carbon and nitrogen stable isotope values (determined using EA-IRMS, see next section in Supplementary Text 5). Collagen yield was consistently above the cut-off of 1 %, ranging from 4 to 12.6 %, and elemental concentrations (%C: 44.1–46.3 %; %N: 16.1–16.9 %) were close to those expected for pristine collagen[103]. C/N ratios all fell between 3.2 and 3.3 (accepted values 2.9 to 3.3[103]). This indicates that collagen extracts contained well-preserved collagen and excludes contamination with hydrocarbon based consolidants such as paraffin, which have been documented in some specimens of the Hülle faunal collection.

Six extracts were sent to the Curt-Engelhorn-Center for Archaeometry in Mannheim, Germany (CEZA, laboratory code: MAMS), where the collagen was combusted to CO_2 in an elemental analyzer (EA) and converted catalytically to graphite, before being measured on a MICADAS AMS[104]. Ten extracts were sent to the Laboratory of Ion Beam Physics at ETH Zürich, Switzerland (laboratory code: ETH) where they were graphitised using the AGE III system (Automated Graphitisation Equipment[105]) and ^{14}C-dated using a MICADAS AMS[106]. Aliquots of a background bone (>50,000 BP) were pretreated and dated alongside the equid samples to monitor lab-based contamination and were used in the age calculation of the samples. Data reduction was performed using BATS software[107] and an additional 1 ‰ was added to the error calculation of the samples, as per standard practice. The ^{14}C dates were calibrated in OxCal 4.4[108] using the IntCal20 calibration curve[109]. Uncalibrated ^{14}C dates (^{14}C BP) are reported with their 1σ error and calibrated ranges (cal BP) are reported at the 2σ range (95 % probability). All dates have been rounded to the nearest 10 years.

5.5 Carbon and nitrogen stable isotope analysis

Carbon and nitrogen isotopic analyses were conducted using a Flash 2000 Organic Elemental Analyzer (Thermo Fisher Scientific, Bremen, Germany) coupled to a Delta XP ratio mass spectrometer (Thermo Fisher Scientific, Bremen, Germany) via a Conflo III interface (Thermo Fisher Scientific, Bremen, Germany). Approximately 0.5 mg of collagen was weighed into tin capsules and introduced to the Elemental Analyzer using an AS 200S autosampler. The oxidation and reduction reactors were held at 1020 °C and 650 °C respectively. Gas separation was achieved with the GC column held at 65 °C with a carrier gas flow of 115 mL/min. All samples were analysed in duplicate with an average precision of replicate measurements of 0.09 ‰ for δ^{13}C and 0.04 ‰ for δ^{15}N. Carbon and nitrogen stable isotope values were two-point scale normalised to the VPDB and AIR scale respectively using IAEA-CH-6 (sucrose, δ^{13}C = -10.449 $\pm$ 0.033 ‰), IAEA-CH-7 (polyethylene, δ^{13}C = -32.151 $\pm$ 0.050 ‰), IAEA-N-1 (ammonium sulphate, δ^{15}N = 0.4 $\pm$ 0.2 ‰) and IAEA-N-2 (ammonium sulphate, δ^{15}N = 20.3 $\pm$ 0.2 ‰). Normalisation was checked using an in-house methionine standard (EVA-0012; n = 8; Elemental Microanalysis, Okehampton, UK) which gave values of -28.05 $\pm$ 0.09 ‰ (1 s.d.) for δ^{13}C and -6.43 $\pm$ 0.03 ‰ (1 s.d) for δ^{15}N and an in-house gelatine standard (EVA MRG, n = 8), which gave values of -19.84 $\pm$ 0.11 ‰ (1 s.d.) for δ^{13}C and 4.95 $\pm$ 0.10 ‰ (1 s.d) for δ^{15}N. This compares well to their long-term average values of -28.0 $\pm$ 0.1 ‰ (1 s.d) (EVA-0012) and -19.7 $\pm$ 0.3 ‰ (1 s.d) (EVA MRG) for δ^{13}C and -6.4 $\pm$ 0.1 ‰ (1 s.d) (EVA-0012) and 5.0 $\pm$ 0.1 ‰ (1 s.d) (EVA MRG) for δ^{15}N.

5.6 Inverse modelling

Prior to summer and winter palaeotemperature estimation, $\delta^{18}O_{phos}$ time series were processed using an inverse model following ref.[110], to remove the damping effect of amelogenesis and the sampling methodology on the seasonal δ^{18}O amplitude. This method

estimates the original $\delta^{18}O$ input into tooth enamel using specifics of the sampling geometry and species-specific parameters of enamel formation fed into an iterative inverse model that mathematically simulates the time-averaging introduced through enamel mineralisation and the sampling procedure. A detailed description of the modelling procedure with associated MATLAB code can be found in ref.@Passey2005a. In this study, an implementation of the original code in R, modified after ref.[111] was used, which can be found at https://osf.io/wunfd/. A more detailed code documentation with example data can also be found at https://github.com/scpederzani/Oxygen_Inverse_Model. It should be noted that this inverse model was developed for applications to ever-growing teeth and does not take into account the progressive slowing of enamel growth towards the enamel-root-junction (ERJ) seen in some molars of large ungulates, particularly late erupting teeth of horses[112,113]. For this reason, updated inverse models have been published for sheep[114] but, unfortunately, no such model currently exists for horses. Due to this effect, the seasonal amplitudes reconstructed here may represent minimum amplitudes.

Model input parameters were chosen to reflect *Equus* sp. enamel formation, following values given in ref.[115] and ref.[116]. The initial mineral content of enamel was input as 22%, and length of apposition and maturation as 6 mm and 28 mm respectively. Sample input variables were given for distance between samples and sample depth. During the modelling process a damping factor characterising the damping of the oxygen isotope time series amplitude needs to be determined using an adjustment of a measured error term (E_{meas}) to the prediction error (E_{pred}). The damping factors of the specimens analysed in this study ranged between 0.0005 and 0.14. Input and derived parameters used for each specimen can be seen in the model reports included in the associated online repository https://osf.io/wunfd/. The repository also includes graphical representations of all model outcomes.

For each specimen the most likely model solution was extracted and used to derive corrected summer peak and winter trough $\delta^{18}O$ values. Corrected summer and winter values were extracted in the $\delta^{18}O$ time series locations that correspond to peaks and troughs in the original unmodelled $\delta^{18}O$ seasonal profile. Corrected summer peak and winter trough values were used as the input for estimating seasonal $\delta^{18}O_{dw}$ values as well as summer and winter temperatures. Mean annual estimates were derived directly from raw $\delta^{18}O_{phos}$ values, rather than from model corrected curves. This approach was taken because the inverse model symmetrically extends the amplitude of the sinusoidal $\delta^{18}O$ curve if a systematic sampling geometry was used, and therefore has very little impact on mean $\delta^{18}O$ of an annual cycle (see ref.@pederzani2021 for a detailed discussion). Annual averages were calculated as the mean of the summer peak and winter trough $\delta^{18}O$ values of each specimen. If several years were represented in a single tooth, multiple annual averages were computed from the different summer/winter combinations. This approach was used following recommendations in ref.[72], which showed no significant differences between such two-point annual averages and means of full annual cycles.

5.7 Palaeotemperature estimation

Palaeotemperatures were computed following methods outlined in ref.[26] using an R implementation of the mathematical equations and excel templates for computations of temperature estimates and associated uncertainties published therein. Mathematical specifications can be obtained from ref.[26] or by consulting the temperature estimation R script, which is included in the online data repository of this study (https://osf.io/wunfd/). Enamel $\delta^{18}O$ to $\delta^{18}O_{dw}$ regressions used in this study were established using ordinary least square regression (OLS) applied to published data for equids refs.[117–120]. Modern calibration data to establish the relationship between precipitation $\delta^{18}O$ and air temperature was obtained from GNIP stations[121] in Europe (exclud-

ing Iberia) and northern Asia to obtain as much temperature coverage as possible, particularly for low temperature environments. Included GNIP stations comprise locations in the following countries: Austria, Belarus, Croatia, Czech Republic, Estonia, Finland, France, Georgia, Germany, Greece, Greenland, Iceland, Italy, Latvia, Mongolia, Norway, Poland, Republic of Moldova, Romania, Russian Federation, Slovakia, Slovenia, Sweden, Ukraine, United Kingdom. If possible, only stations with five or more years of measurements were used. Separate calibration data sets were used for summer, winter and mean annual calibrations. This is firstly because the slope of the $\delta^{18}O_{precip}$ - air temperature relationship is known to vary seasonally, but also because some GNIP stations only report measurements for certain months of the year. These separate data sets were compiled from GNIP data for the warmest month (commonly July or August; $n_{stations}$ = 113), coldest month (commonly December or January; $n_{stations}$ = 112) and long-term mean annual averages respectively ($n_{stations}$ = 90). Regression lines for the $\delta^{18}O_{precip}$ - air temperature relationships were calculated using OLS. All calibration data sets are available in the electronic data repository of this study at https://osf.io/wunfd/.

Following the workflow presented in ref.[26], palaeotemperature estimates are normally made as an average for each stratigraphic unit, with data from all specimens from the same layer being combined into one temperature estimate. As the equid data used here relies on direct radiocarbon dates for chronological control, we binned the $\delta^{18}O$ data of specimens according to data clusters seen in radiocarbon dates and $\delta^{18}O$ data, with most time bins covering ~2000 years (Supplementary Table 6). This binning of data enables the use of a sample size dependent calculation of estimation uncertainty but introduces a degree of averaging of between-year climatic variability and biological or behavioural variability between individuals. For this reason, true temperature variability is likely underestimated in the palaeotemperature data as a certain degree of time-averaging is introduced in the palaeotemperature estimates. Additionally, temperature

seasonality derived from such pooled summer and winter temperature estimates may deviate somewhat from the full extent of annual temperature fluctuation that may have occurred in some years.

5.8 Software and code

This article, including code for all data analyses, was written in R (version 4.2.0)[122] on a Windows 10 operating system and the manuscript and supplementary information rendered using Quarto[123]. The code for data analysis to render the article manuscript and supplementary information rendering makes use of the following packages: cowplot[124], flextable[125], ggbeeswarm[126], ggcorrplot[127], ggpattern[128], ggpubr[129], ggrepel[130], knitr[131], magick[132], MetBrewer[133], officer[134], scales[135], tidyverse[136].

Data and code to reproduce the manuscript files, figures and analyses are available at https://osf.io/wunfd/. More throuroughly documented and clean example scripts and files for the inverse model and the temperature estimation can be additionally be found at https://github.com/scpederzani/Oxygen_Inverse_Model and https://github.com/scpederzani/Isotope_Tempe

6 Supplementary Figures

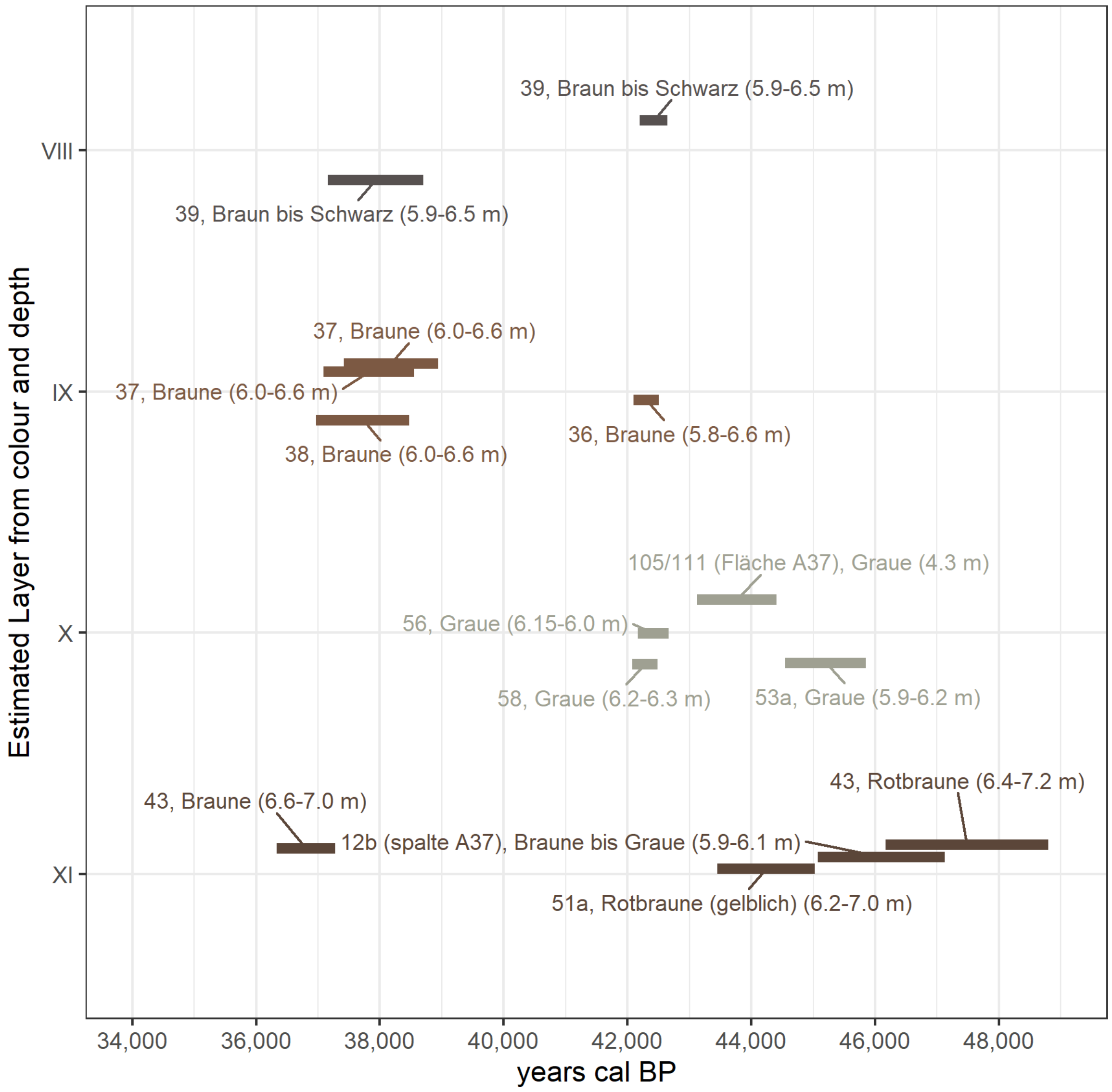

Supplementary Figure 1: Plot of direct radiocarbon dates of equid teeth in comparison to the layer that was assigned based on the description of layer colour and depth on find boxes. Depth was correlated with nearest available profile drawing, but use of depths was complicated by pronounced depth fluctuations within the site and by mismatches of depth measurements on profile drawings between original excavation diaries and the site monograph. The comparison of layers assigned in this manner with the radiocarbon dates of equid specimens shows that this process often led to misassignments especially for the different brown layers (IX and XI), where layer color could not reliably be used to differentiate the strata. Layer labels are given as written on find boxes (Braun = brown, Schwarz = black, Grau = grey, Rotbraun/Rot braun = red brown, gelblich = yellowish). Depth information as labelled on find boxes is given in parentheses.

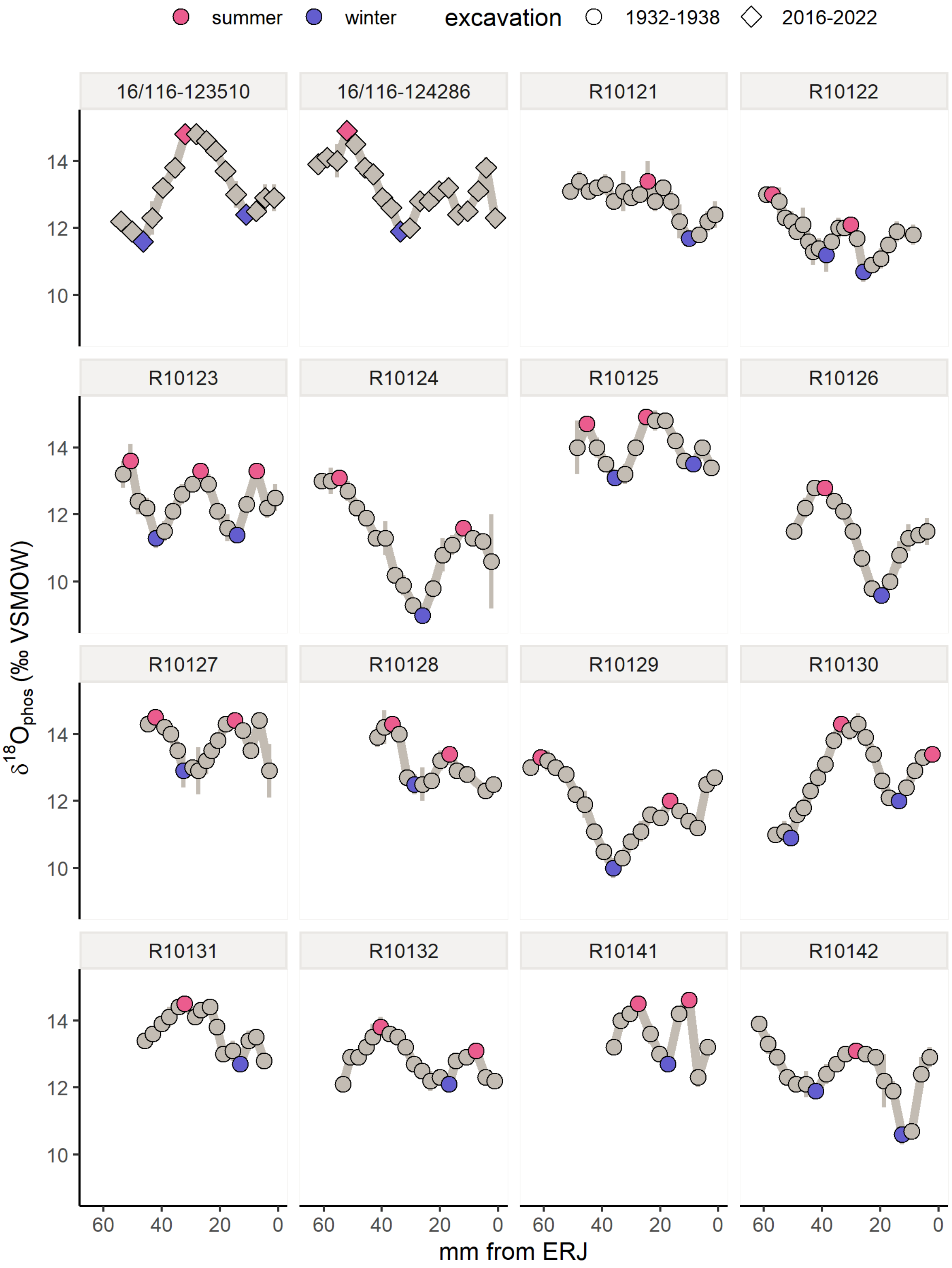

Supplementary Figure 2: Plot of oxygen stable isotope time series data for each tooth ordered by find number. Peaks of sinusoidal curves represent summer season input, while troughs form during the winter season. Colour-marked summer (pink) and winter (purple) season measurements were extracted to investigate diachronic change of seasonal climatic conditions. Oxygen isotope measurements are plotted against distance from the enamel-root-junction (ERJ). Stable isotope data are presented as the mean ± analytical reproducibility (1 s.d. of n = 3 replicate sample measurements).

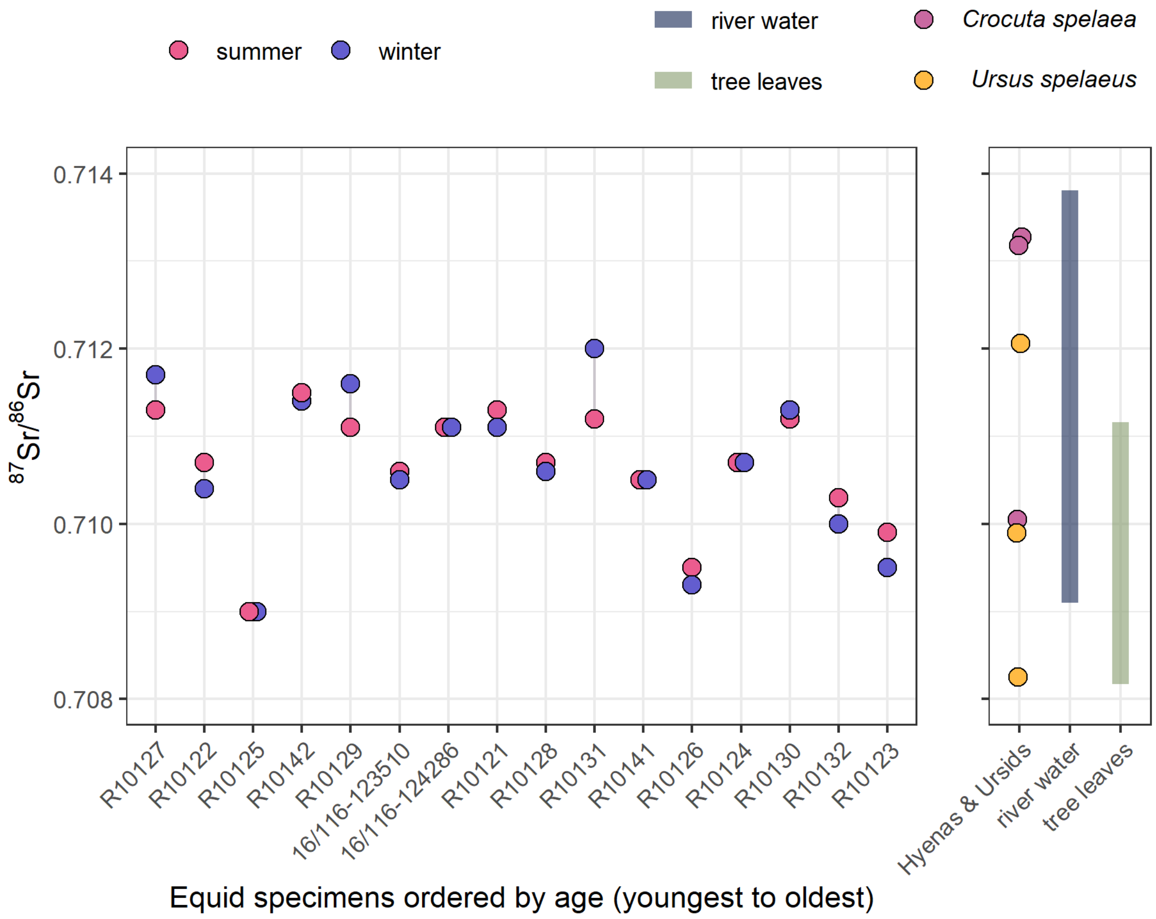

Supplementary Figure 3: Summer (pink) and winter (purple) $^{87}Sr/^{86}Sr$ values of equids (left) fall into the value range of bioavailable Sr in eastern Thuringia (right, green = tree leaf samples[65]; blue = water samples from the upper course of the Saale River[67]). Similarly, equid $^{87}Sr/^{86}Sr$ matches values of hyenas and ursids that typically have small to modest range sizes in the Pleistocene (right). These overlaps indicate that equids most likely ranged across lithologies within a few tens of kilometres of Ranis. This is supported by small summer/winter value differences in almost all cases, with the exception of specimen R10131, which shows a seasonal $^{87}Sr/^{86}Sr$ amplitude of almost a full third decimal. A migratory ecology remains unlikely but cannot be excluded for this individual. Summer and winter season for the equid samples were determined using oxygen stable isotopes (see Supplementary Figure 2). Note that taxonomic carnivorans are not necessarily dietary carnivores but can be omnivores with a largely plant-based diet. Analytical reproducibility is smaller than point size.

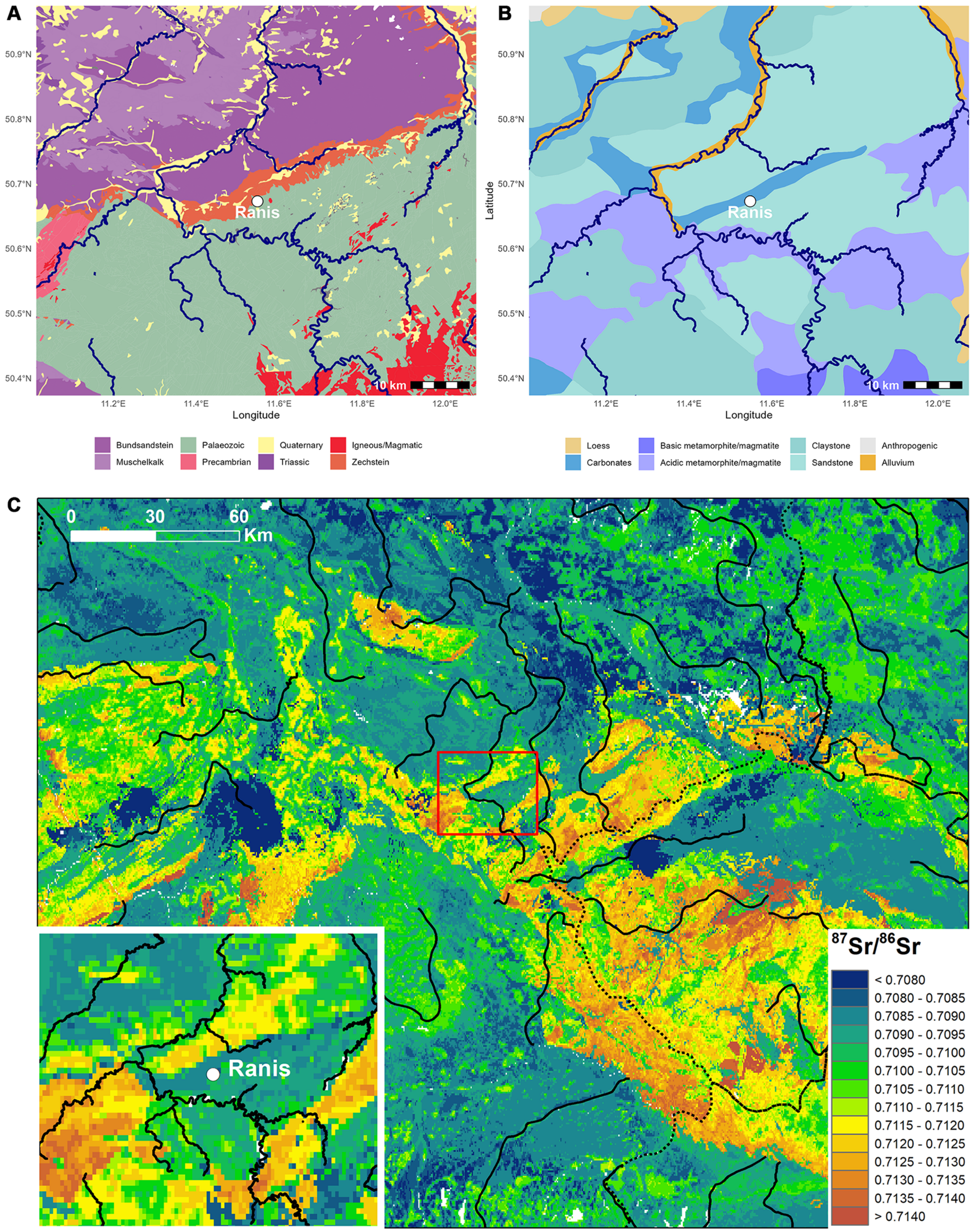

Supplementary Figure 4: Map of bedrock geological units (A), soil parent bedrock types (B), and predicted $^{87}Sr/^{86}Sr$ values (C) in the study area around Ranis. Bedrock geological units were obtained from DTK200 leaf numbers GK5534, GK5526, GK6334, GK6326[137], parent bedrock types were obtained from BAG5000[138]. Muschelkalk and Buntsandstein are Triassic formations, while the Zechstein formation is a Permian (Palaeozoic) formation that includes prominent carbonate formations. Igneous lithologies are from the Upper Devonian (Palaeozoic). Colors of geological units follow conventions based in unit age, with the exception of igneous lithologies that are colored red according to rock type conventions. The predicted isoscape of bioavailable strontium isotopic ratios (C) were derived from the global strontium isoscape in ref.[139].

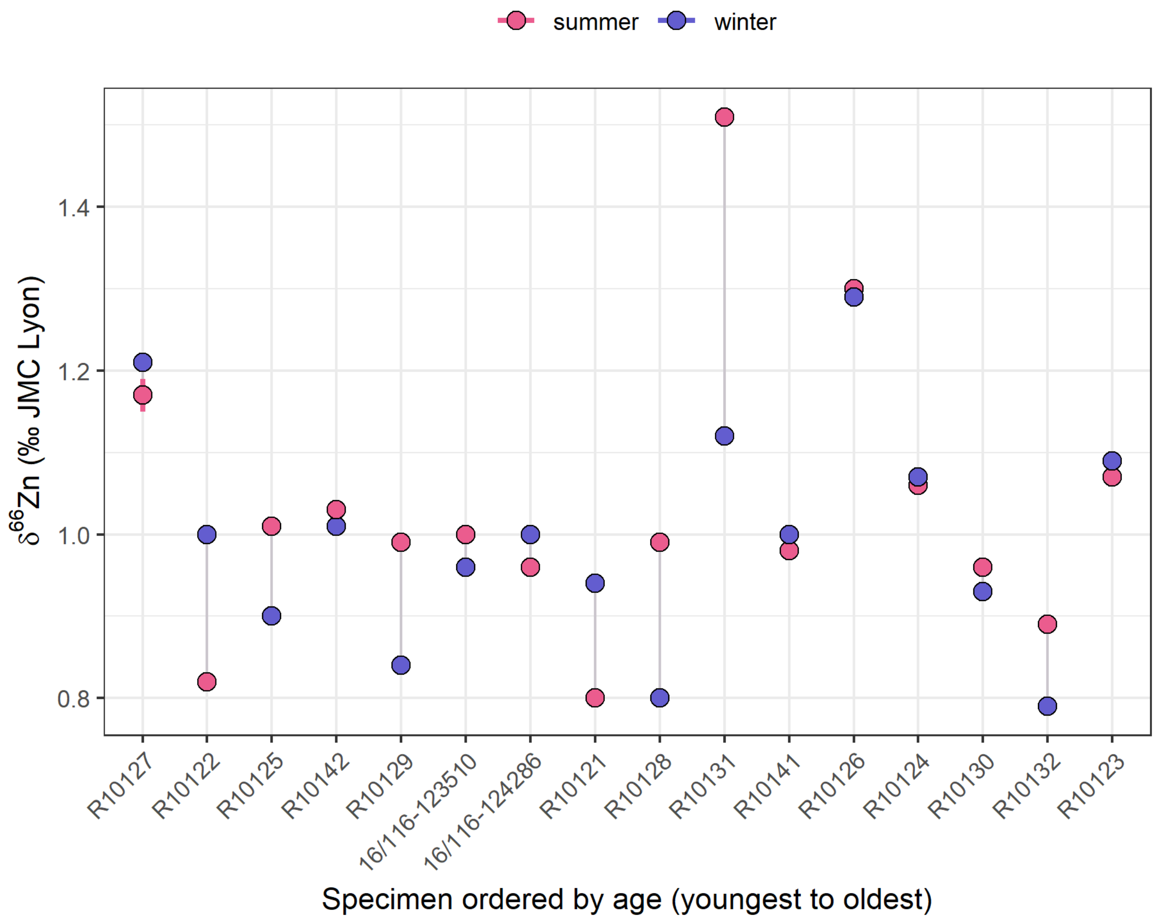

Supplementary Figure 5: Difference between summer (pink) and winter (purple) enamel δ^{66}Zn values of equids are small (< 0.2 ‰) in most specimens, with the exception of specimen R10131, which shows a seasonal δ^{66}Zn amplitude of ~0.4 ‰, larger than a typical trophic level spacing. Based on comparison with ^{87}Sr/^{86}Sr values we suggest that this may be linked to seasonal ranging across different lithologies. The direction of summer/winter δ^{66}Zn difference does not show an apparent pattern, but small absolute seasonal amplitudes are mostly observed in specimens with high δ^{66}Zn, perhaps indicating a higher degree of dietary specialisation in these individuals. Error bars represent 1 s.d. of duplicate measurements but are smaller than symbol size in most cases.

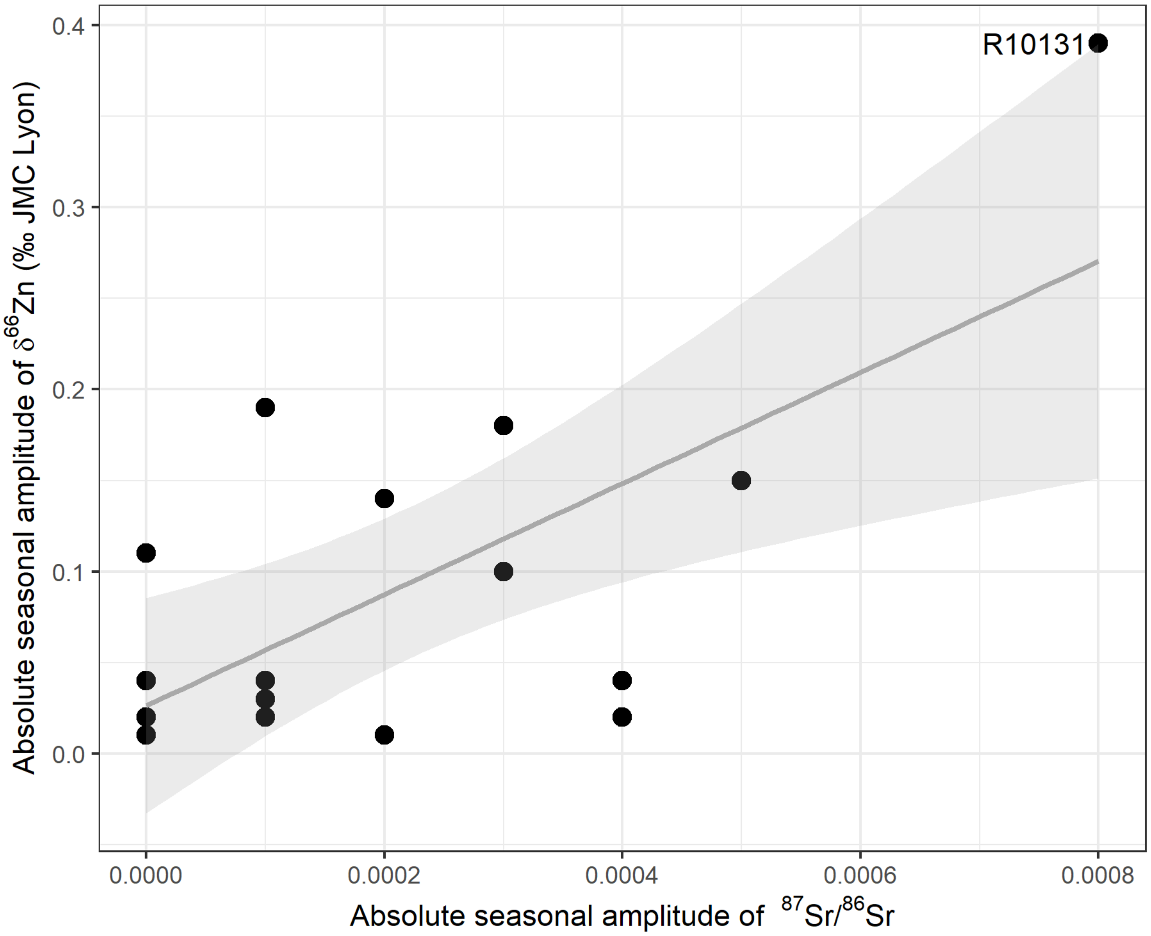

Supplementary Figure 6: Equid specimen R10131 shows an uncommonly large absolute seasonal amplitude (magnitude of summer-winter difference) in both zinc and strontium isotope compositions. Any correlation between zinc and strontium isotope absolute seasonal amplitude is only driven by this outlier specimen, underlining that other specimens show a secure dietary pattern in zinc stable isotope compositions. For R10131, its outlier position might indicate that this individual seasonally ranged over isotopically distinct lithologies. While we argue that long-distance migratory behaviour is still unlikely for this individual, it cannot be fully excluded, and we therefore remove this specimen from climatic interpretations of oxygen stable isotopes and dietary interpretations of zinc stable isotopes. The shaded area marks the 95% confidence interval of the OLS linear regression.

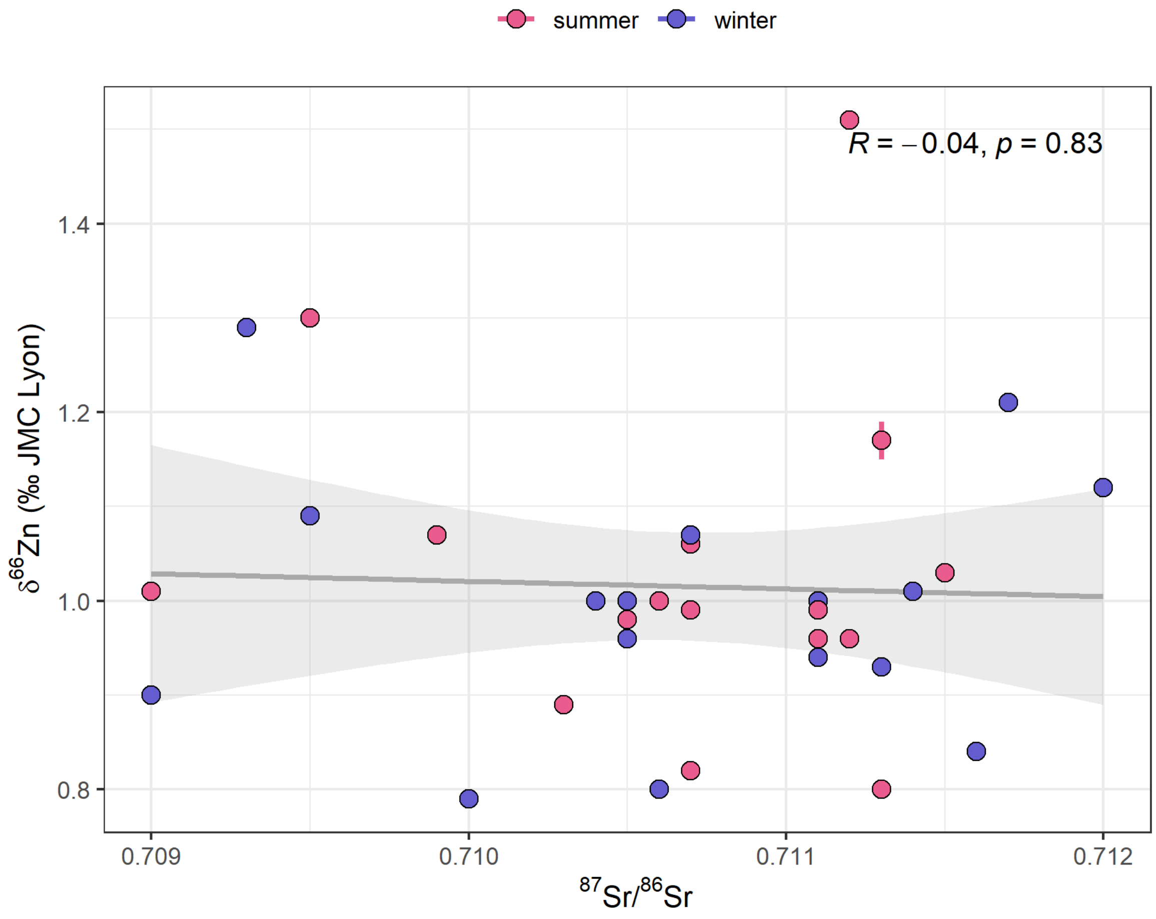

Supplementary Figure 7: Equid samples do not show a correlation between δ^{66}Zn and ^{87}Sr/^{86}Sr, indicating that δ^{66}Zn is predominantly controlled by dietary ecology or other factors not related to underlying lithology. Correlation calculated using Pearson correlation test. Error bars represent 1 s.d. of duplicate measurements but are often smaller than symbol size.

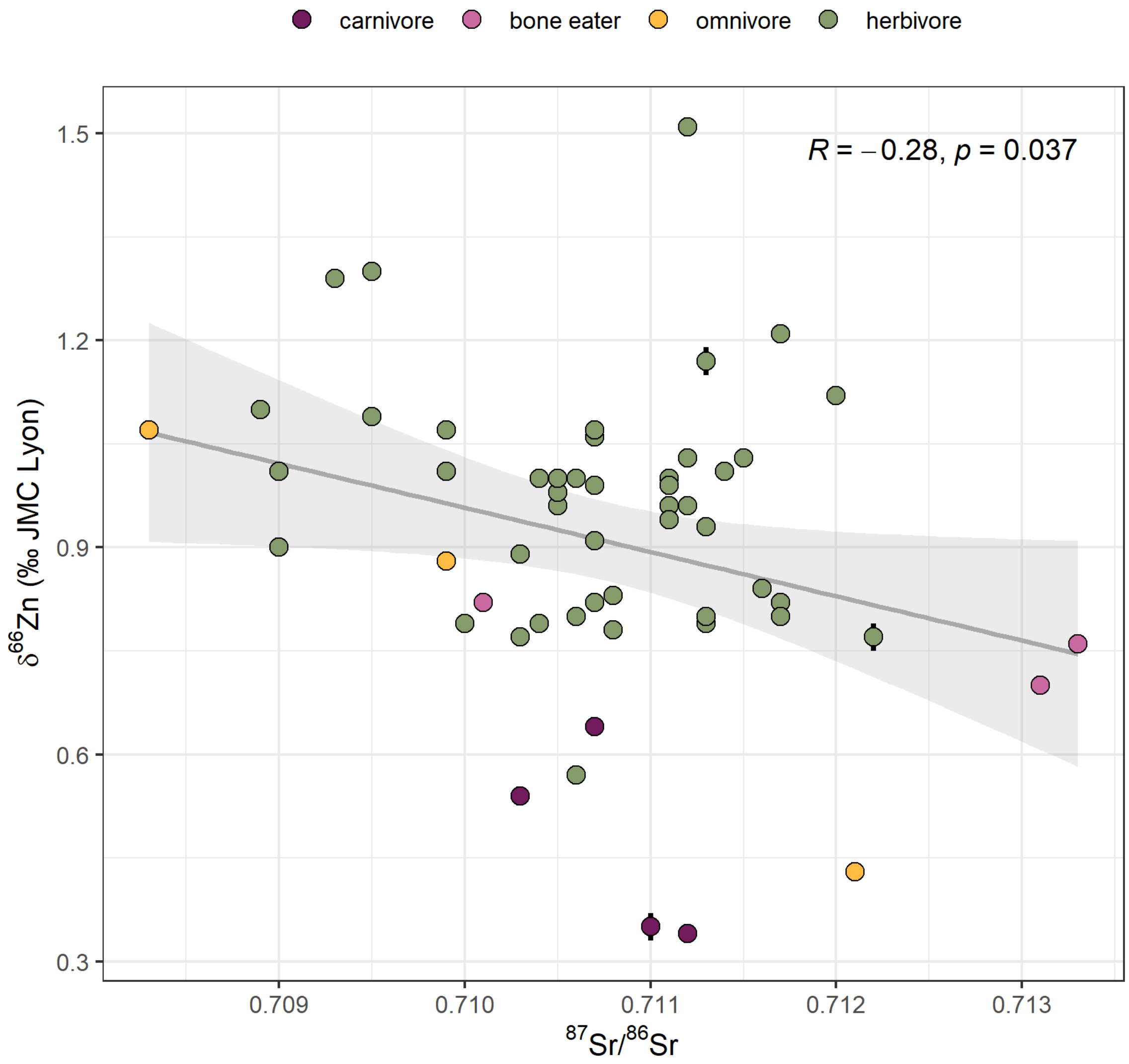

Supplementary Figure 8: Across all taxa sampled from the food web we observe a statistically significant correlation between δ^{66}Zn and ^{87}Sr/^{86}Sr (Pearson correlation test). This may show a small geological influence on δ^{66}Zn. However, the relationship has a very small slope coefficient and appears mostly driven by two hyena samples with higher ^{87}Sr/^{86}Sr (without hyena specimens p = 0.083, Pearson correlation). We therefore argue that diet is the dominant driver of δ^{66}Zn in this food web, while bedrock geology plays a very minor role. Error bars represent 1 s.d. of duplicate measurements but are often smaller than symbol size.

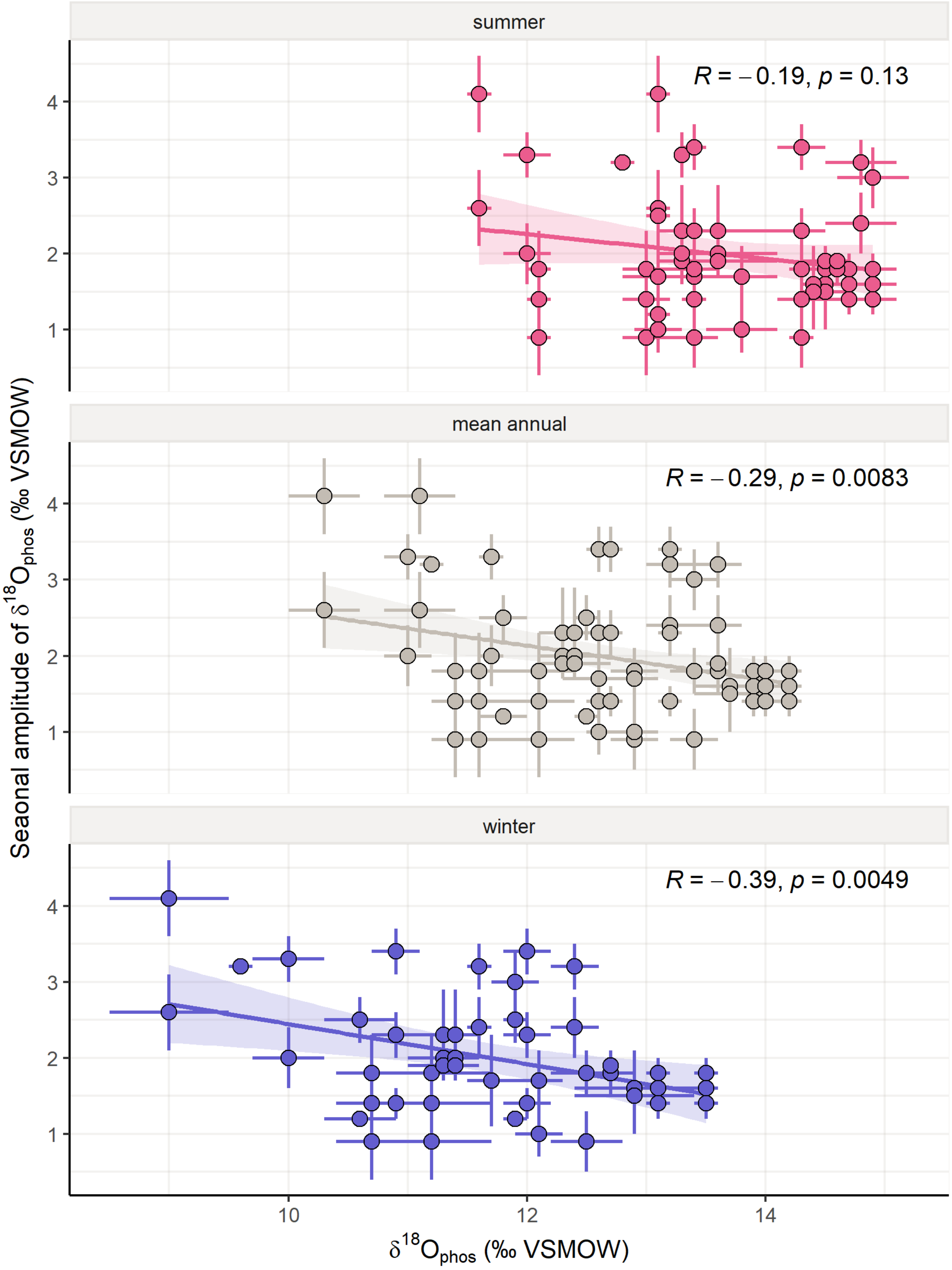

Supplementary Figure 9: Seasonal δ^{18}O amplitudes (summer-winter differences) are lower in specimens with higher δ^{18}O values. This negative correlation is statistically significant for winter δ^{18}O, but not summer δ^{18}O (two-sided Pearson correlation). This means that changes in seasonality are driven predominantly by changes in winter δ^{18}O, while summer δ^{18}O is more stable through time. Stable isotope data are presented as the mean ± analytical reproducibility (1 s.d. of n = 3 replicate sample measurements). Lines and shaded areas represent an ordinary least squares regression line with 96% confidence interval.

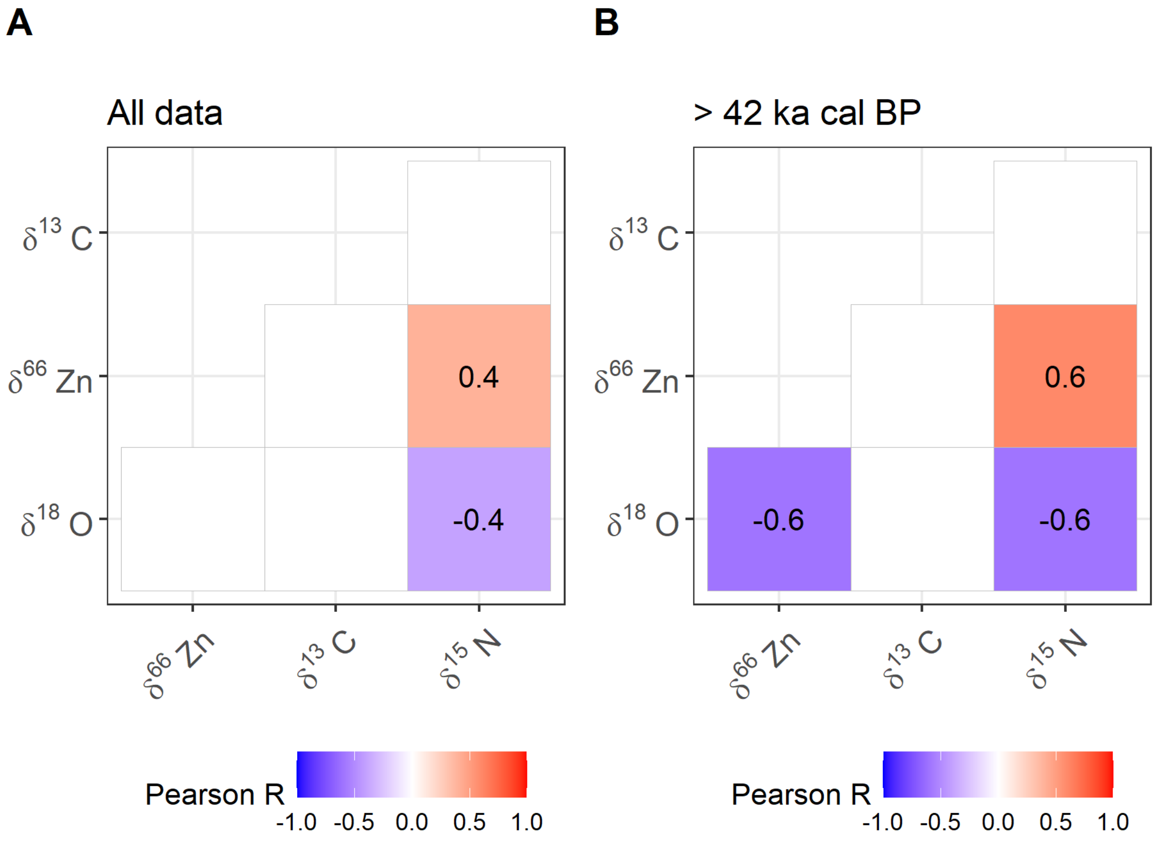

Supplementary Figure 10: A: Across the complete analysed stratigraphic sequence we observe statistically significant correlations between δ^{18}O and δ^{15}N (negative) and between δ^{66}Zn and δ^{15}N (positive, Pearson correlation). B: A negative correlation is also observed between δ^{18}O and δ^{66}Zn during the δ^{18}O decline from ~45-43 ka cal BP. No other isotope proxy correlates with δ^{13}C regardless of time frame.

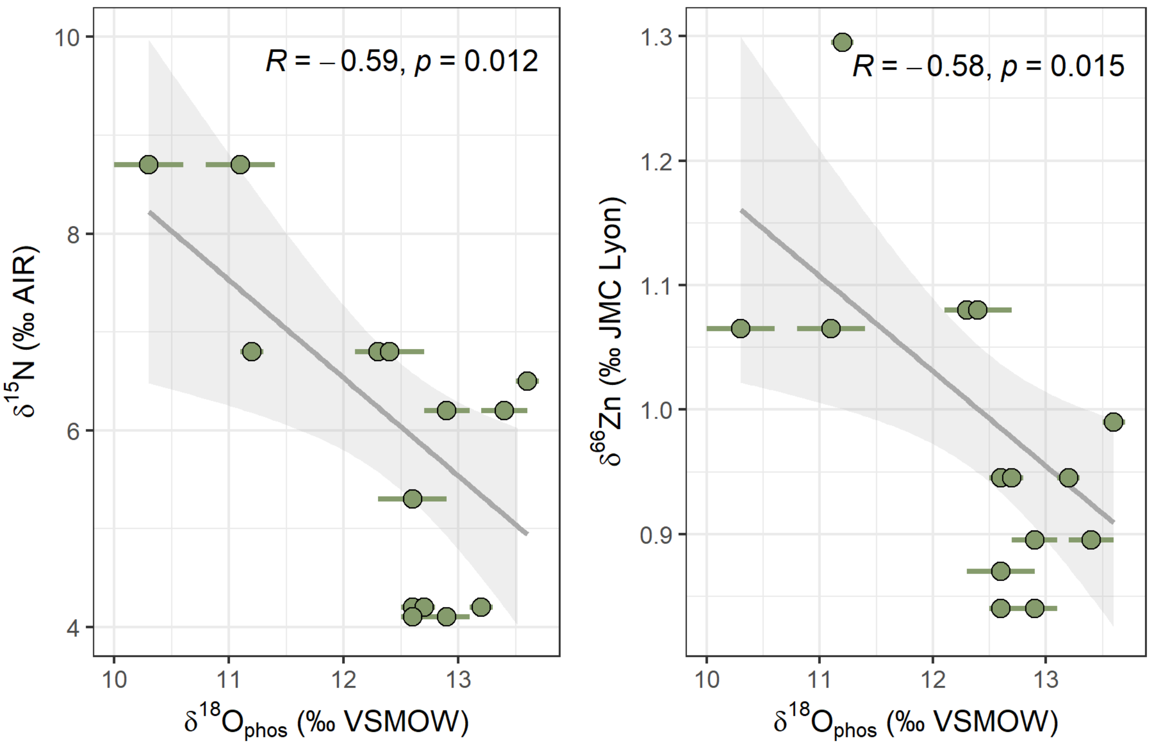

Supplementary Figure 11: The decline of equid δ^{18}O between ~45 and 43 ka cal BP is accompanied by rising δ^{66}Zn and δ^{15}N, causing negative correlations (two-sided Pearson correlation) for these tracer combinations for equid specimens dating to > 42 ka cal BP. Error bars represent analytical reproducibility (1 s.d. of replicate sample measurements) but are often smaller than symbol size. Lines and shaded areas represent an ordinary least squares regression line with 96% confidence interval.

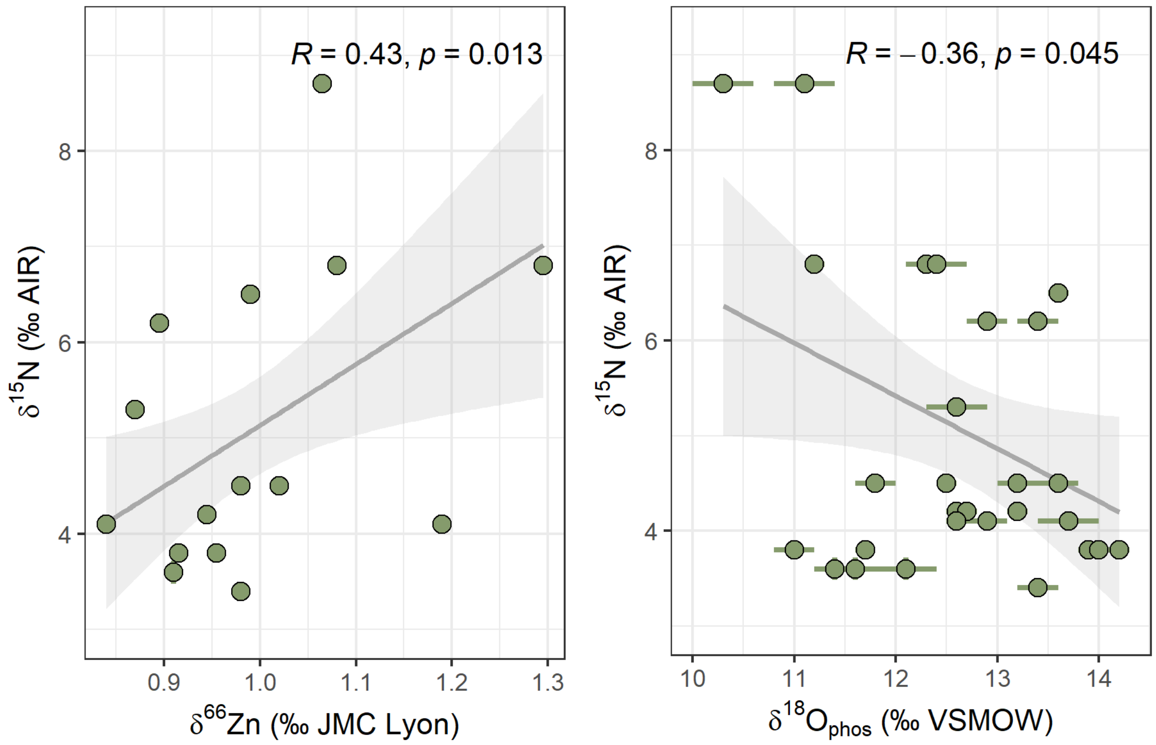

Supplementary Figure 12: In equid specimens from all analysed time phases δ^{18}O correlates negatively with δ^{15}N, while δ^{66}Zn and δ^{15}N correlate positively (two-sided Pearson correlation). Error bars represent analytical reproducibility (1 s.d. of replicate sample measurements) but are often smaller than symbol size. Lines and shaded areas represent an ordinary least squares regression line with 96% confidence interval.

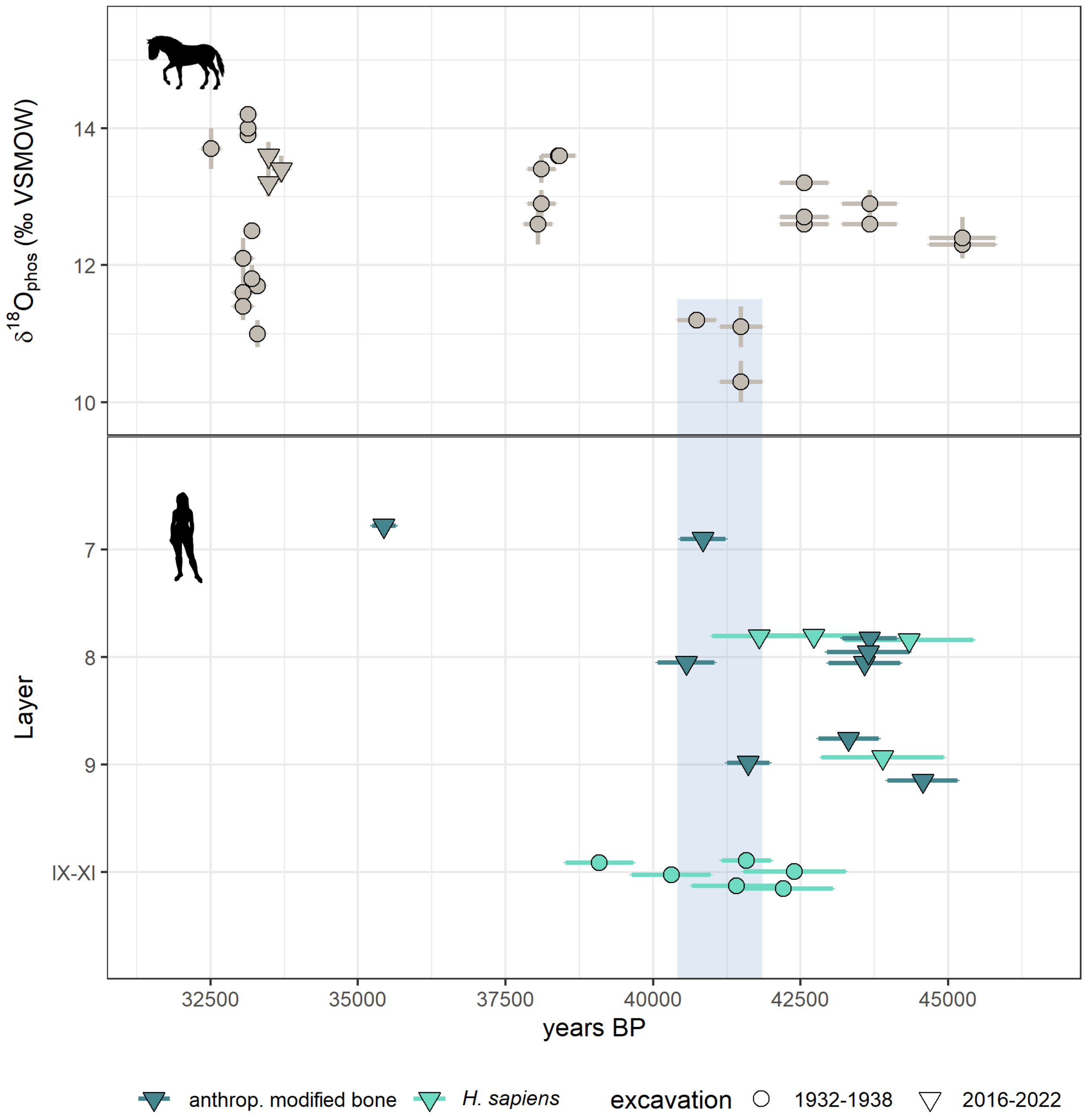

Supplementary Figure 13: Comparison of equid δ^{18}O data (top) with directly dated *H. sapiens* remains[5] (bottom, turquoise points) and faunal bone fragments with anthropogenic surface modifications (dark teal points) demonstrates extensive overlap of *H. sapiens* presence with the coldest temperatures (marked by blue shading) also for uncalibrated dates. Data are presented as mean ± the error of uncalibrated radiocarbon ages (n = 1 bone/tooth sample for each data point), while point shape indicates whether specimens were found in the Hülle (1932–1938, circles) or the TLDA/MPI-EVA (2016–2022, triangles) excavation collection. Vertical error bars represent analytical reproducibility (1 s.d. of n = 3 replicate sample measurements). We argue that *H. sapiens* fragments from the Hülle collection (labelled IX-XI here) all originate from the LRJ deposits (Layer X) and were sometimes assigned to a mixture of Layer X and adjacent strata by the original excavators due to rough excavation methods (see details in ref.[5]). We have pooled all these samples here to reflect this. The human silhouette originates from the voyager plaque (NASA, no copyright), equid silhouette obtained from www.phylopic.org (c Mercedes Yrayzoz/Michael Keesey under cc-by 3.0 https://creativecommons.org/licenses/by/3.0/).

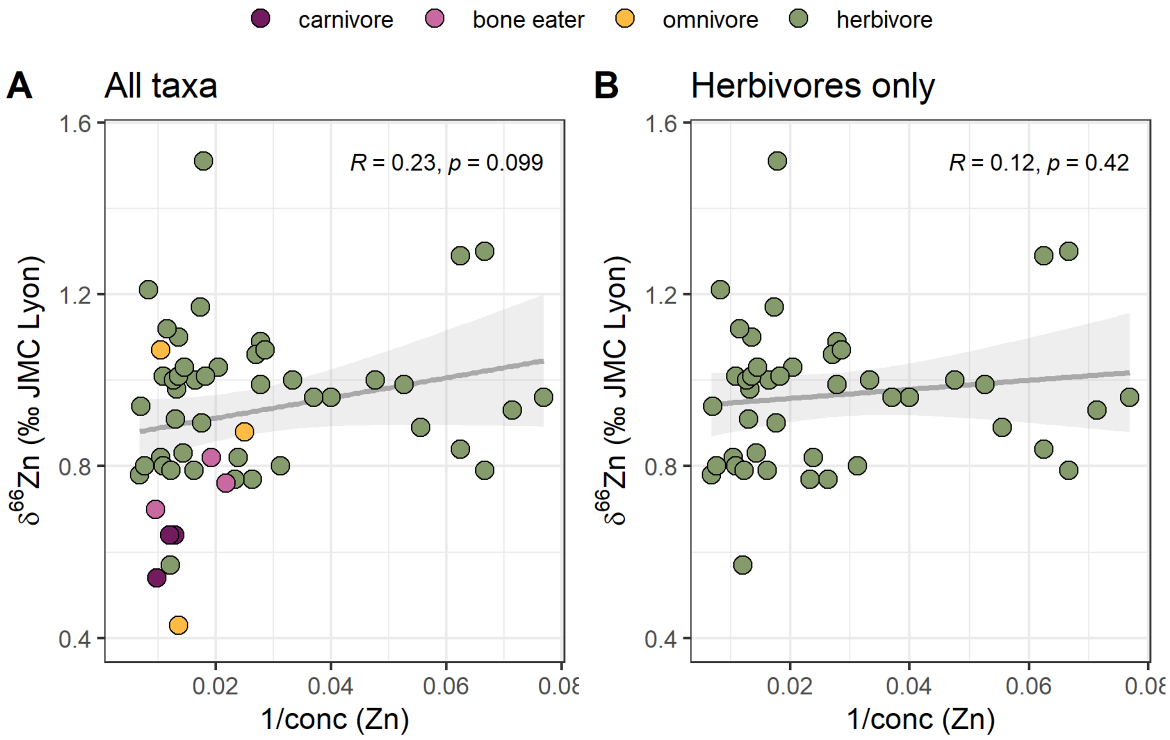

Supplementary Figure 14: A lack of mixing line (correlation coefficients from two-sided Pearson correlation) between δ^{66}Zn and 1/conc (Zn) in the whole food web (A) or herbivores only (B) suggests a lack of zinc uptake from the depositional environment and, thus, preservation of biogenic δ^{66}Zn. We tested both the complete food web and herbivores only to test for statistical artefacts in the whole food web test, that could be caused the much higher herbivore sample size compared to the other dietary groups. Shaded grey error bands represent the 95% confidence interval in both panels.

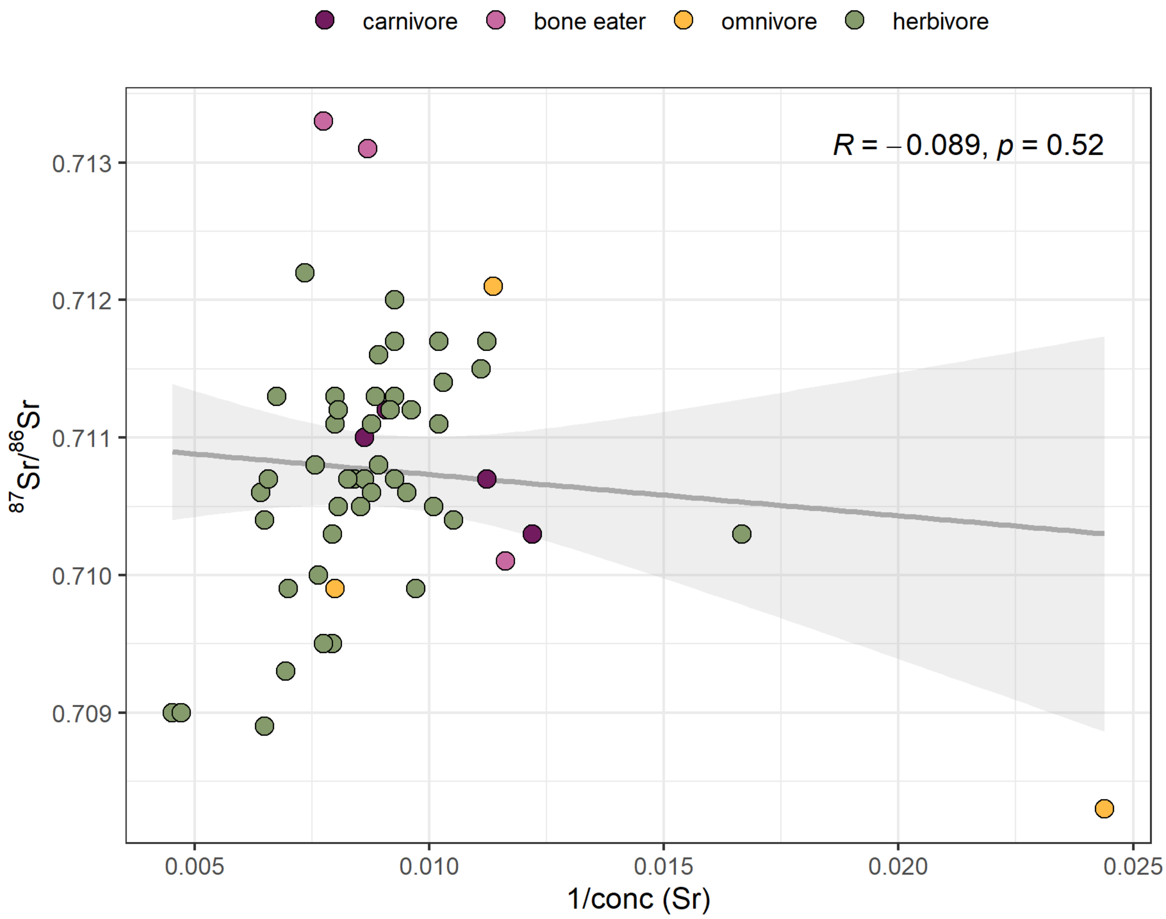

Supplementary Figure 15: A lack of mixing line (correlation coefficients from two-sided Pearson correlation) between $^{87}Sr/^{86}Sr$ and 1/conc (Sr) indicates a lack of uptake of exogenous strontium from the depositional environment and, thus, preservation of biogenic $^{87}Sr/^{86}Sr$. The shaded grey error band represents the 95% confidence interval.

7 Supplementary Tables

Supplementary Table 1: Overview of all specimens that were analysed for this study with contextual and zooarchaeological information. Equids were used for oxygen, carbon, nitrogen, strontium, and zinc stable isotope analysis and were directly radiocarbon dated. All other specimens were only analysed for zinc and strontium stable isotopes.

SQUID	Excavation	Square	Layer	Taxon	Element
16/116-123510	TLDA/MPI-EVA	1004/999	6 (Black)	*Equus ferus*	lower left P3
16/116-124286	TLDA/MPI-EVA	1004/999	6 (Black)	*Equus ferus*	upper right P3
R10121	Hülle	58	Graue (6.2-6.3 m)	*Equus sp.*	upper right P3
R10122	Hülle	38	Braune (6.0-6.6 m)	*Equus ferus*	lower right M3
R10123	Hülle	43	Rotbraune (6.4-7.2 m)	*Equus ferus*	upper left M3
R10124	Hülle	51a	Rotbraune (gelblich) (6.2-7.0 m)	*Equus ferus*	upper left M2
R10125	Hülle	37	Braune (6.0-6.6 m)	*Equus ferus*	upper left P2
R10126	Hülle	105/111	Graue (4.3 m)	*Equus ferus*	lower left M3
R10127	Hülle	43	Braune (6.6-7.0 m)	*Equus ferus*	upper right P3
R10128	Hülle	36	Braune (5.8-6.6 m)	*Equus ferus*	upper left M3
R10129	Hülle	37	Braune (6.0-6.6 m)	*Equus ferus*	lower right M2
R10130	Hülle	53a	Graue (5.9-6.2 m)	*Equus ferus*	lower right P4
R10131	Hülle	56	Graue (6.15-6.0 m)	*Equus ferus*	upper left M2
R10132	Hülle	12b	Braune bis Graue (5.9-6.1 m)	*Equus ferus*	lower right M3
R10141	Hülle	39	Braun bis Schwarz (5.9-6.5 m)	*Equus ferus*	upper right M3
R10142	Hülle	39	Braun bis Schwarz (5.9-6.5 m)	*Equus ferus*	upper right M2
R10148a	Hülle	34	Braune (6.0-7.2 m)	*Cervid*	M1
R10148b	Hülle	34	Braune (6.0-7.2 m)	*Rangifer tarandus*	M3
R10149a	Hülle	36	Braune (5.8-6.6 m)	*Vulpes lagopus*	M2
R10149b	Hülle	36	Braune (5.8-6.6 m)	*Vulpes lagopus*	C
R10150	Hülle	36	Braune (5.8-6.6 m)	*Coelodonta antiquitatis*	dP2

SQUID	Excavation	Square	Layer	Taxon	Element
R10151	Hülle	36	Braune (5.8-6.6 m)	*Cervus elaphus*	M1
R10152	Hülle	36	Braune (5.8-6.6 m)	*Cervid*	P3
R10156	Hülle	21	Braune (5.4-6.0 m)	*Crocuta spelaea*	P3
R10157	Hülle	21	Braune (5.4-6.0 m)	*Crocuta spelaea*	P3
R10158	Hülle	21	Braune (5.4-6.0 m)	*Crocuta spelaea*	M1
R10159	Hülle	38	Braune (6.0-6.6 m)	*Ursus spelaeus*	C
R10160	Hülle	38	Braune (6.0-6.6 m)	*Ursus spelaeus*	dP
R10161	Hülle	38	Braune (6.8-7.2 m)	*Vulpes lagopus*	premolar
R10162	Hülle	38	Braune (7.2-7.7 m)	*Coelodonta antiquitatis*	molar
R10163	Hülle	38	Braune (5.8-7.7)	*Cervus elaphus*	P4
R10164	Hülle	38	Braune (5.8-7.7)	*Vulpes lagopus*	M1
R10165	Hülle	37	Braune (6.0-6.6 m)	*Cervus elaphus*	M3
R10166	Hülle	37	Braune (6.0-6.6 m)	*Cervus elaphus*	P4
R10167	Hülle	43/44	Braune (7.2-7.6 m)	*Cervid*	P4
R10168	Hülle	44	Braune (6.6-7.0 m)	*Coelodonta antiquitatis*	M1
R10169	Hülle	44	Braune (6.6-7.0 m)	*Cervid*	molar
R10170	Hülle	40/42	Braune (6.7-7.2 m)	*carnivore small*	I3
R10171	Hülle	40/42	Braune (6.7-7.2 m)	*Ursus spelaeus*	C
R10172	Hülle	40/42	Braune (6.4-7.2 m)	*Rangifer tarandus*	P2, P3

Supplementary Table 2: Results of direct radiocarbon dating of equid specimens including quality control indicators (collagen yield, elemental composition, C/N ratio, carbon and nitrogen stable isotope values). Information on tooth position and contextual information for each specimen can be found in Supplementary Table 1.

SQUID	Material	AMS Code	% yield	% C	% N	C/N	$\delta^{13}C$	SD	$\delta^{15}N$	SD	^{14}C age	error	yrs cal BP (68%)	yrs cal BP (95%)
16/116-123510	mandible bone	ETH-111914	12.1	45.7	16.6	3.2	-20.6	0.04	4.5	0.04	33,480	140	38,860 – 37,810	39,080 – 37,640
16/116-124286	mandible bone	ETH-111915	12.6	45.3	16.4	3.2	-20.9	0.04	3.4	0.00	33,700	150	39,180 – 38,500	39,290 – 37,880
R10121	dentine	ETH-111919	10.1	46.3	16.8	3.2	-21.2	0.01	5.3	0.02	38,050	240	42,390 – 42,190	42,490 – 42,080
R10122	dentine	MAMS-42387	11.8	44.9	16.2	3.2	-20.7	0.13	3.6	0.12	33,050	170	37,760 – 37,080	38,470 – 36,970
R10123	dentine	ETH-111910	8.6	46.2	16.9	3.2	-20.7	0.05	6.8	0.01	45,240	560	48,170 – 46,870	48,800 – 46,170
R10124	dentine	ETH-111922	5.3	44.8	16.4	3.2	-21.8	0.36	8.7	0.08	41,490	360	44,690 – 44,160	45,020 – 43,440
R10125	dentine	ETH-111911	11.8	45.3	16.5	3.2	-20.9	0.01	3.8	0.03	33,140	140	38,070 – 37,290	38,550 – 37,090
R10126	dentine	ETH-111920	5.8	45.4	16.5	3.2	-20.7	0.01	6.8	0.00	40,740	330	44,150 – 43,360	44,400 – 43,120
R10127	dentine	MAMS-42389	12.2	46.3	16.6	3.3	-20.8	0.36	4.1	0.07	32,510	170	37,030 – 36,570	37,280 – 36,340
R10128	dentine	ETH-111912	8.1	44.8	16.3	3.2	-20.8	0.07	6.2	0.02	38,110	240	42,410 – 42,210	42,510 – 42,100
R10129	dentine	ETH-111913	7.7	46.0	16.6	3.2	-21.0	0.01	3.8	0.03	33,300	140	38,370 – 37,580	38,940 – 37,420
R10130	dentine	ETH-111921	4.0	44.4	16.2	3.2	-21.3	0.01	4.2	0.01	42,560	410	45,440 – 44,750	45,850 – 44,550
R10131	dentine	MAMS-42388	7.9	45.2	16.3	3.2	-21.1	0.29	7.2	0.06	38,400	290	42,520 – 42,280	42,670 – 42,170

SQUID	Material	AMS Code	% yield	% C	% N	C/N	$\delta^{13}C$	SD	$\delta^{15}N$	SD	^{14}C age	error	yrs cal BP (68%)	yrs cal BP (95%)
R10132	dentine	ETH-111918	5.0	44.5	16.2	3.2	-21.2	0.01	4.1	0.05	43,670	460	46,490 – 45,500	47,130 – 45,080
R10141	dentine	ETH-111916	8.0	45.8	16.6	3.2	-20.9	0.03	6.5	0.02	38,410	250	42,510 – 42,300	42,640 – 42,200
R10142	dentine	ETH-111917	6.4	44.1	16.1	3.2	-20.8	0.07	4.5	0.04	33,200	140	38,230 – 37,450	38,700 – 37,160

Supplementary Table 3: Summary statistics (means, standard deviation, maxima, minima) of unmodelled seasonal and mean annual oxygen stable isotope values for each time bin that was used to reconstruct palaeotemperature estimates. Summary statistics are also provided for seasonal amplitudes (summer-winter differences). Number of data points can vary between different seasons of the same time bin, as oxygen isotope time series often include multiple or incomplete years and therefore can contain more summer than winter data points and vice versa.

Age bin (cal BP)	Record type	mean $\delta^{18}O_{phos}$	SD	min $\delta^{18}O_{phos}$	max $\delta^{18}O_{phos}$	$N_{datapoints}$
36000 - 39000	summer	13.8	1.1	12.0	14.9	11
42000 - 43000	summer	14.0	0.6	13.4	14.6	5
43000 - 45000	summer	12.5	0.8	11.6	13.1	3
45000 - 48000	summer	13.5	0.4	13.1	14.3	7
36000 - 39000	mean annual	12.8	1.1	11.0	14.2	15
42000 - 43000	mean annual	13.2	0.4	12.6	13.6	5
43000 - 45000	mean annual	10.9	0.5	10.3	11.2	3
45000 - 48000	mean annual	12.6	0.3	12.3	13.2	9
36000 - 39000	winter	11.8	1.1	10.0	13.5	11
42000 - 43000	winter	12.3	0.5	11.7	12.7	3
43000 - 45000	winter	9.3	0.4	9.0	9.6	2
45000 - 48000	winter	11.5	0.5	10.9	12.1	5
36000 - 39000	seasonal amplitude	2.0	0.7	0.9	3.3	15

Age bin (cal BP)	Record type	mean $\delta^{18}O_{phos}$	SD	min $\delta^{18}O_{phos}$	max $\delta^{18}O_{phos}$	$N_{datapoints}$
42000 - 43000	seasonal amplitude	1.6	0.4	0.9	1.9	5
43000 - 45000	seasonal amplitude	3.3	0.8	2.6	4.1	3
45000 - 48000	seasonal amplitude	2.0	0.7	1.0	3.4	9

Supplementary Table 4: Individual oxygen stable isotope measurements obtained from sequentially sampled tooth enamel.

Sample ID	SQUID	Taxon	mm from ERJ	mean $\delta^{18}O_{phos}$	SD
R10121.A	R10121	*Equus sp.*	50.8	13.1	0.2
R10121.B	R10121	*Equus sp.*	47.8	13.4	0.3
R10121.C	R10121	*Equus sp.*	44.6	13.1	0.2
R10121.D	R10121	*Equus sp.*	41.7	13.2	0.2
R10121.E	R10121	*Equus sp.*	38.9	13.3	0.3
R10121.F	R10121	*Equus sp.*	35.9	12.8	0.2
R10121.G	R10121	*Equus sp.*	32.7	13.1	0.6
R10121.H	R10121	*Equus sp.*	30.0	12.9	0.2
R10121.I	R10121	*Equus sp.*	27.1	13.0	0.2
R10121.J	R10121	*Equus sp.*	24.3	13.4	0.6
R10121.K	R10121	*Equus sp.*	21.7	12.8	0.3
R10121.L	R10121	*Equus sp.*	19.1	13.2	0.2
R10121.M	R10121	*Equus sp.*	16.2	12.8	0.3
R10121.N	R10121	*Equus sp.*	13.4	12.2	0.5
R10121.O	R10121	*Equus sp.*	10.0	11.7	0.2
R10121.P	R10121	*Equus sp.*	6.7	11.8	0.2
R10121.Q	R10121	*Equus sp.*	3.8	12.2	0.3
R10121.R	R10121	*Equus sp.*	1.2	12.4	0.4
R10122.A	R10122	*Equus ferus*	59.2	13.0	0.0
R10122.B	R10122	*Equus ferus*	57.2	13.0	0.2
R10122.C	R10122	*Equus ferus*	54.8	12.8	0.4
R10122.D	R10122	*Equus ferus*	52.7	12.3	0.1
R10122.E	R10122	*Equus ferus*	50.6	12.2	0.3
R10122.F	R10122	*Equus ferus*	48.8	11.9	0.1
R10122.G	R10122	*Equus ferus*	46.5	12.1	0.5
R10122.H	R10122	*Equus ferus*	44.7	11.6	0.2
R10122.I	R10122	*Equus ferus*	43.1	11.3	0.4
R10122.J	R10122	*Equus ferus*	41.1	11.4	0.3
R10122.K	R10122	*Equus ferus*	38.5	11.2	0.5
R10122.L	R10122	*Equus ferus*	36.7	11.6	0.1
R10122.M	R10122	*Equus ferus*	34.4	12.0	0.3
R10122.N	R10122	*Equus ferus*	32.4	12.0	0.0
R10122.O	R10122	*Equus ferus*	30.2	12.1	0.1

Sample ID	SQUID	Taxon	mm from ERJ	mean $\delta^{18}O_{phos}$	SD
R10122.P	R10122	*Equus ferus*	28.0	11.7	0.2
R10122.Q	R10122	*Equus ferus*	25.8	10.7	0.3
R10122.R	R10122	*Equus ferus*	23.0	10.9	0.1
R10122.S	R10122	*Equus ferus*	19.8	11.1	0.3
R10122.T	R10122	*Equus ferus*	17.3	11.5	0.2
R10122.U	R10122	*Equus ferus*	14.3	11.9	0.3
R10122.V	R10122	*Equus ferus*	8.7	11.8	0.3
R10122.W	R10122	*Equus ferus*	4.5	13.2	0.2
R10123.A	R10123	*Equus ferus*	53.3	13.2	0.4
R10123.B	R10123	*Equus ferus*	50.7	13.6	0.5
R10123.C	R10123	*Equus ferus*	48.1	12.4	0.4
R10123.D	R10123	*Equus ferus*	45.0	12.2	0.3
R10123.E	R10123	*Equus ferus*	42.0	11.3	0.3
R10123.F	R10123	*Equus ferus*	39.0	11.5	0.3
R10123.G	R10123	*Equus ferus*	36.2	12.1	0.2
R10123.H	R10123	*Equus ferus*	33.0	12.6	0.3
R10123.I	R10123	*Equus ferus*	29.5	12.9	0.2
R10123.J	R10123	*Equus ferus*	26.7	13.3	0.1
R10123.K	R10123	*Equus ferus*	23.9	12.9	0.3
R10123.L	R10123	*Equus ferus*	20.8	12.1	0.1
R10123.M	R10123	*Equus ferus*	17.4	11.6	0.4
R10123.N	R10123	*Equus ferus*	14.2	11.4	0.1
R10123.O	R10123	*Equus ferus*	10.9	12.3	0.2
R10123.P	R10123	*Equus ferus*	7.3	13.3	0.1
R10123.Q	R10123	*Equus ferus*	3.8	12.2	0.3
R10123.R	R10123	*Equus ferus*	1.1	12.5	0.4
R10124.A	R10124	*Equus ferus*	60.6	13.0	0.2
R10124.B	R10124	*Equus ferus*	57.6	13.0	0.4
R10124.C	R10124	*Equus ferus*	54.7	13.1	0.1
R10124.D	R10124	*Equus ferus*	51.7	12.7	0.3
R10124.E	R10124	*Equus ferus*	48.7	12.2	0.2
R10124.F	R10124	*Equus ferus*	45.4	11.9	0.1
R10124.G	R10124	*Equus ferus*	42.0	11.3	0.1
R10124.H	R10124	*Equus ferus*	38.8	11.3	0.5
R10124.I	R10124	*Equus ferus*	35.5	10.2	0.1

Sample ID	SQUID	Taxon	mm from ERJ	mean $\delta^{18}O_{phos}$	SD
R10124.J	R10124	*Equus ferus*	32.6	9.9	0.3
R10124.K	R10124	*Equus ferus*	29.4	9.3	0.2
R10124.L	R10124	*Equus ferus*	26.1	9.0	0.5
R10124.M	R10124	*Equus ferus*	22.4	9.8	0.3
R10124.N	R10124	*Equus ferus*	19.1	10.8	0.5
R10124.O	R10124	*Equus ferus*	15.9	11.1	0.3
R10124.P	R10124	*Equus ferus*	12.1	11.6	0.1
R10124.Q	R10124	*Equus ferus*	8.7	11.3	0.2
R10124.R	R10124	*Equus ferus*	5.3	11.2	0.1
R10124.S	R10124	*Equus ferus*	2.4	10.6	1.4
R10125.A	R10125	*Equus ferus*	51.2	14.1	0.2
R10125.B	R10125	*Equus ferus*	48.6	14.0	0.8
R10125.C	R10125	*Equus ferus*	45.2	14.7	0.2
R10125.D	R10125	*Equus ferus*	41.7	14.0	0.3
R10125.E	R10125	*Equus ferus*	38.6	13.5	0.2
R10125.F	R10125	*Equus ferus*	35.6	13.1	0.1
R10125.G	R10125	*Equus ferus*	32.1	13.2	0.3
R10125.H	R10125	*Equus ferus*	28.4	14.0	0.2
R10125.I	R10125	*Equus ferus*	24.9	14.9	0.2
R10125.J	R10125	*Equus ferus*	21.7	14.8	0.3
R10125.K	R10125	*Equus ferus*	18.2	14.8	0.2
R10125.L	R10125	*Equus ferus*	14.9	14.2	0.2
R10125.M	R10125	*Equus ferus*	11.7	13.6	0.2
R10125.N	R10125	*Equus ferus*	8.6	13.5	0.1
R10125.O	R10125	*Equus ferus*	5.5	14.0	0.2
R10125.P	R10125	*Equus ferus*	2.5	13.4	0.2
R10126.A	R10126	*Equus ferus*	49.7	11.5	0.1
R10126.B	R10126	*Equus ferus*	45.9	12.2	0.3
R10126.C	R10126	*Equus ferus*	42.5	12.8	0.1
R10126.D	R10126	*Equus ferus*	39.1	12.8	0.1
R10126.E	R10126	*Equus ferus*	35.9	12.4	0.2
R10126.F	R10126	*Equus ferus*	32.7	12.1	0.1
R10126.G	R10126	*Equus ferus*	29.4	11.5	0.1
R10126.H	R10126	*Equus ferus*	26.4	10.7	0.2
R10126.I	R10126	*Equus ferus*	23.0	9.8	0.2

Sample ID	SQUID	Taxon	mm from ERJ	mean $\delta^{18}O_{phos}$	SD
R10126.J	R10126	*Equus ferus*	19.7	9.6	0.1
R10126.K	R10126	*Equus ferus*	16.8	10.0	0.1
R10126.L	R10126	*Equus ferus*	13.4	10.8	0.4
R10126.M	R10126	*Equus ferus*	10.3	11.3	0.4
R10126.N	R10126	*Equus ferus*	6.9	11.4	0.0
R10126.O	R10126	*Equus ferus*	4.0	11.5	0.4
R10126.P	R10126	*Equus ferus*	1.1	12.9	0.2
R10127.A	R10127	*Equus ferus*	44.7	14.3	0.2
R10127.B	R10127	*Equus ferus*	42.2	14.5	0.2
R10127.C	R10127	*Equus ferus*	39.0	14.2	0.2
R10127.D	R10127	*Equus ferus*	36.9	14.0	0.1
R10127.E	R10127	*Equus ferus*	34.5	13.5	0.1
R10127.F	R10127	*Equus ferus*	32.5	12.9	0.5
R10127.G	R10127	*Equus ferus*	29.4	13.0	0.1
R10127.H	R10127	*Equus ferus*	27.5	12.9	0.7
R10127.I	R10127	*Equus ferus*	24.6	13.2	0.4
R10127.J	R10127	*Equus ferus*	22.9	13.5	0.1
R10127.K	R10127	*Equus ferus*	20.6	13.8	0.2
R10127.L	R10127	*Equus ferus*	18.0	14.3	0.1
R10127.M	R10127	*Equus ferus*	14.9	14.4	0.1
R10127.N	R10127	*Equus ferus*	12.1	14.1	0.2
R10127.O	R10127	*Equus ferus*	9.3	13.5	0.0
R10127.P	R10127	*Equus ferus*	6.4	14.4	0.1
R10127.Q	R10127	*Equus ferus*	3.0	12.9	0.8
R10128.A	R10128	*Equus ferus*	41.6	13.9	0.3
R10128.B	R10128	*Equus ferus*	39.1	14.2	0.5
R10128.C	R10128	*Equus ferus*	36.5	14.3	0.1
R10128.D	R10128	*Equus ferus*	34.1	14.0	0.3
R10128.E	R10128	*Equus ferus*	31.4	12.7	0.2
R10128.F	R10128	*Equus ferus*	28.8	12.5	0.3
R10128.G	R10128	*Equus ferus*	26.0	12.5	0.5
R10128.H	R10128	*Equus ferus*	23.0	12.6	0.2
R10128.I	R10128	*Equus ferus*	19.8	13.2	0.3
R10128.J	R10128	*Equus ferus*	16.7	13.4	0.2
R10128.K	R10128	*Equus ferus*	14.2	12.9	0.2

Sample ID	SQUID	Taxon	mm from ERJ	mean $\delta^{18}O_{phos}$	SD
R10128.L	R10128	*Equus ferus*	10.7	12.8	0.2
R10128.M	R10128	*Equus ferus*	7.4	14.6	0.4
R10128.N	R10128	*Equus ferus*	4.3	12.3	0.1
R10128.O	R10128	*Equus ferus*	1.6	12.5	0.1
R10129.A	R10129	*Equus ferus*	64.5	13.0	0.2
R10129.B	R10129	*Equus ferus*	61.1	13.3	0.0
R10129.C	R10129	*Equus ferus*	58.7	13.2	0.3
R10129.D	R10129	*Equus ferus*	56.0	13.0	0.0
R10129.E	R10129	*Equus ferus*	52.3	12.8	0.1
R10129.F	R10129	*Equus ferus*	49.0	12.2	0.2
R10129.G	R10129	*Equus ferus*	46.0	11.9	0.4
R10129.H	R10129	*Equus ferus*	42.7	11.1	0.1
R10129.I	R10129	*Equus ferus*	39.4	10.5	0.2
R10129.J	R10129	*Equus ferus*	36.2	10.0	0.3
R10129.K	R10129	*Equus ferus*	33.1	10.3	0.1
R10129.L	R10129	*Equus ferus*	30.1	10.8	0.2
R10129.M	R10129	*Equus ferus*	26.6	11.1	0.3
R10129.N	R10129	*Equus ferus*	23.3	11.6	0.0
R10129.O	R10129	*Equus ferus*	20.0	11.5	0.0
R10129.P	R10129	*Equus ferus*	16.7	12.0	0.2
R10129.Q	R10129	*Equus ferus*	13.4	11.7	0.1
R10129.R	R10129	*Equus ferus*	10.3	11.4	0.0
R10129.S	R10129	*Equus ferus*	7.1	11.2	0.2
R10129.T	R10129	*Equus ferus*	4.1	12.5	0.1
R10129.U	R10129	*Equus ferus*	1.3	12.7	0.0
R10130.A	R10130	*Equus ferus*	56.0	11.0	0.1
R10130.B	R10130	*Equus ferus*	53.0	11.1	0.3
R10130.C	R10130	*Equus ferus*	50.7	10.9	0.2
R10130.D	R10130	*Equus ferus*	48.6	11.6	0.0
R10130.E	R10130	*Equus ferus*	46.4	11.8	0.2
R10130.F	R10130	*Equus ferus*	44.0	12.3	0.1
R10130.G	R10130	*Equus ferus*	41.5	12.7	0.3
R10130.H	R10130	*Equus ferus*	38.9	13.1	0.1
R10130.I	R10130	*Equus ferus*	36.1	13.8	0.1
R10130.J	R10130	*Equus ferus*	33.5	14.3	0.2

Sample ID	SQUID	Taxon	mm from ERJ	mean $\delta^{18}O_{phos}$	SD
R10130.K	R10130	*Equus ferus*	30.6	14.1	0.3
R10130.L	R10130	*Equus ferus*	27.6	14.3	0.3
R10130.M	R10130	*Equus ferus*	25.1	13.9	0.2
R10130.N	R10130	*Equus ferus*	22.3	13.4	0.2
R10130.O	R10130	*Equus ferus*	19.5	12.6	0.1
R10130.P	R10130	*Equus ferus*	17.0	12.1	0.2
R10130.Q	R10130	*Equus ferus*	13.7	12.0	0.2
R10130.R	R10130	*Equus ferus*	11.0	12.4	0.1
R10130.S	R10130	*Equus ferus*	8.1	12.9	0.2
R10130.T	R10130	*Equus ferus*	5.4	13.3	0.2
R10130.U	R10130	*Equus ferus*	2.1	13.4	0.1
R10131.A	R10131	*Equus ferus*	45.8	13.4	0.1
R10131.B	R10131	*Equus ferus*	43.0	13.6	0.1
R10131.C	R10131	*Equus ferus*	40.0	13.9	0.1
R10131.D	R10131	*Equus ferus*	37.4	14.1	0.3
R10131.E	R10131	*Equus ferus*	34.1	14.4	0.2
R10131.F	R10131	*Equus ferus*	32.2	14.5	0.2
R10131.G	R10131	*Equus ferus*	28.6	14.1	0.2
R10131.H	R10131	*Equus ferus*	26.5	14.3	0.1
R10131.I	R10131	*Equus ferus*	23.5	14.4	0.1
R10131.J	R10131	*Equus ferus*	21.1	13.8	0.1
R10131.K	R10131	*Equus ferus*	18.8	13.0	0.1
R10131.L	R10131	*Equus ferus*	15.6	13.1	0.3
R10131.M	R10131	*Equus ferus*	13.1	12.7	0.2
R10131.N	R10131	*Equus ferus*	10.1	13.4	0.3
R10131.O	R10131	*Equus ferus*	7.6	13.5	0.1
R10131.P	R10131	*Equus ferus*	4.8	12.8	0.2
R10131.Q	R10131	*Equus ferus*	1.7	9.8	0.1
R10132.A	R10132	*Equus ferus*	53.4	12.1	0.2
R10132.B	R10132	*Equus ferus*	50.8	12.9	0.2
R10132.C	R10132	*Equus ferus*	48.1	12.9	0.2
R10132.D	R10132	*Equus ferus*	45.4	13.2	0.2
R10132.E	R10132	*Equus ferus*	43.1	13.5	0.4
R10132.F	R10132	*Equus ferus*	40.5	13.8	0.3
R10132.G	R10132	*Equus ferus*	37.3	13.6	0.2

Sample ID	SQUID	Taxon	mm from ERJ	mean $\delta^{18}O_{phos}$	SD
R10132.H	R10132	*Equus ferus*	34.6	13.5	0.1
R10132.I	R10132	*Equus ferus*	31.8	13.2	0.1
R10132.J	R10132	*Equus ferus*	28.9	12.7	0.2
R10132.K	R10132	*Equus ferus*	26.2	12.5	0.0
R10132.L	R10132	*Equus ferus*	23.3	12.2	0.3
R10132.M	R10132	*Equus ferus*	20.0	12.3	0.2
R10132.N	R10132	*Equus ferus*	17.0	12.1	0.2
R10132.O	R10132	*Equus ferus*	14.5	12.8	0.1
R10132.P	R10132	*Equus ferus*	11.0	12.9	0.2
R10132.Q	R10132	*Equus ferus*	7.6	13.1	0.2
R10132.R	R10132	*Equus ferus*	4.3	12.3	0.1
R10132.S	R10132	*Equus ferus*	1.2	12.2	0.1
R10141.A	R10141	*Equus ferus*	36.0	13.2	0.0
R10141.B	R10141	*Equus ferus*	33.5	14.0	0.1
R10141.C	R10141	*Equus ferus*	30.4	14.2	0.1
R10141.D	R10141	*Equus ferus*	27.5	14.5	0.2
R10141.E	R10141	*Equus ferus*	23.4	13.6	0.3
R10141.F	R10141	*Equus ferus*	20.3	13.0	0.2
R10141.G	R10141	*Equus ferus*	17.3	12.7	0.0
R10141.H	R10141	*Equus ferus*	13.5	14.2	0.1
R10141.I	R10141	*Equus ferus*	10.1	14.6	0.1
R10141.J	R10141	*Equus ferus*	7.0	12.3	0.3
R10141.K	R10141	*Equus ferus*	3.8	13.2	0.2
R10142.A	R10142	*Equus ferus*	68.3	14.8	0.1
R10142.B	R10142	*Equus ferus*	65.1	14.6	0.1
R10142.C	R10142	*Equus ferus*	61.7	13.9	0.2
R10142.D	R10142	*Equus ferus*	58.7	13.3	0.3
R10142.E	R10142	*Equus ferus*	55.5	12.9	0.2
R10142.F	R10142	*Equus ferus*	52.1	12.3	0.2
R10142.G	R10142	*Equus ferus*	48.9	12.1	0.1
R10142.H	R10142	*Equus ferus*	45.5	12.1	0.4
R10142.I	R10142	*Equus ferus*	42.2	11.9	0.1
R10142.J	R10142	*Equus ferus*	38.6	12.4	0.3
R10142.K	R10142	*Equus ferus*	35.1	12.7	0.2
R10142.L	R10142	*Equus ferus*	32.0	13.0	0.1

Sample ID	SQUID	Taxon	mm from ERJ	mean $\delta^{18}O_{phos}$	SD
R10142.M	R10142	*Equus ferus*	28.3	13.1	0.1
R10142.N	R10142	*Equus ferus*	25.1	13.0	0.2
R10142.O	R10142	*Equus ferus*	21.6	12.9	0.1
R10142.P	R10142	*Equus ferus*	18.7	12.2	0.8
R10142.Q	R10142	*Equus ferus*	15.7	11.9	0.3
R10142.R	R10142	*Equus ferus*	12.6	10.6	0.3
R10142.S	R10142	*Equus ferus*	9.2	10.7	0.1
R10142.T	R10142	*Equus ferus*	5.9	12.4	0.5
R10142.U	R10142	*Equus ferus*	3.1	12.9	0.3
124286.A	16/116-124286	*Equus ferus*	68.7	12.3	0.3
124286.B	16/116-124286	*Equus ferus*	65.2	13.2	0.2
124286.C	16/116-124286	*Equus ferus*	62.0	13.9	0.1
124286.D	16/116-124286	*Equus ferus*	58.8	14.1	0.2
124286.E	16/116-124286	*Equus ferus*	55.4	14.0	0.5
124286.F	16/116-124286	*Equus ferus*	52.0	14.9	0.3
124286.G	16/116-124286	*Equus ferus*	49.1	14.5	0.1
124286.H	16/116-124286	*Equus ferus*	45.8	13.8	0.3
124286.I	16/116-124286	*Equus ferus*	42.9	13.6	0.3
124286.J	16/116-124286	*Equus ferus*	39.8	12.9	0.2
124286.K	16/116-124286	*Equus ferus*	36.8	12.6	0.2
124286.L	16/116-124286	*Equus ferus*	33.6	11.9	0.2
124286.M	16/116-124286	*Equus ferus*	30.4	12.0	0.1
124286.N	16/116-124286	*Equus ferus*	27.0	12.8	0.3
124286.O	16/116-124286	*Equus ferus*	23.8	12.8	0.2
124286.P	16/116-124286	*Equus ferus*	20.4	13.1	0.2
124286.Q	16/116-124286	*Equus ferus*	17.2	13.2	0.3
124286.R	16/116-124286	*Equus ferus*	13.9	12.4	0.2
124286.S	16/116-124286	*Equus ferus*	10.5	12.5	0.3
124286.T	16/116-124286	*Equus ferus*	6.9	13.1	0.2
124286.U	16/116-124286	*Equus ferus*	4.2	13.8	0.3
124286.V	16/116-124286	*Equus ferus*	1.0	12.3	0.1
123510.A	16/116-123510	*Equus ferus*	54.0	12.2	0.2
123510.B	16/116-123510	*Equus ferus*	50.2	11.9	0.2
123510.C	16/116-123510	*Equus ferus*	46.4	11.6	0.1
123510.D	16/116-123510	*Equus ferus*	43.3	12.3	0.5

Sample ID	SQUID	Taxon	mm from ERJ	mean $\delta^{18}O_{phos}$	SD
123510.E	16/116-123510	*Equus ferus*	39.6	13.2	0.2
123510.F	16/116-123510	*Equus ferus*	35.5	13.8	0.2
123510.G	16/116-123510	*Equus ferus*	32.0	14.8	0.3
123510.H	16/116-123510	*Equus ferus*	28.1	14.8	0.7
123510.I	16/116-123510	*Equus ferus*	24.6	14.6	0.1
123510.J	16/116-123510	*Equus ferus*	21.4	14.3	0.3
123510.K	16/116-123510	*Equus ferus*	18.1	13.7	0.3
123510.L	16/116-123510	*Equus ferus*	14.5	13.0	0.4
123510.M	16/116-123510	*Equus ferus*	11.0	12.4	0.2
123510.N	16/116-123510	*Equus ferus*	7.5	12.5	0.3
123510.O	16/116-123510	*Equus ferus*	4.5	12.9	0.4
123510.P	16/116-123510	*Equus ferus*	1.4	12.9	0.4

Supplementary Table 5: Zinc and strontium isotope measurements obtained from a selection of equid enamel samples, representing summer and winter input respectively as well as for bulk samples obtained for a range of carnivore, omnivore, and herbivore species. Samples with multiple letter codes (e.g., 'J+I') indicate that adjacent samples were combined to obtain sufficient material for analysis.

SQUID	Sample ID	Taxon	Record type	$^{87}Sr/^{86}Sr$	Sr conc (ppm)	$\delta^{66}Zn$	SD	Zn conc (ppm)
16/116-123510	123510.H	*Equus sp.*	summer	0.7106	105	1.00	0.01	21
16/116-123510	123510.M	*Equus sp.*	winter	0.7105	99	0.96	0.00	25
16/116-124286	124286.F	*Equus sp.*	summer	0.7111	98	0.96	-	27
16/116-124286	124286.L	*Equus sp.*	winter	0.7111	114	1.00	0.01	61
R10121	R10121.J+I	*Equus sp.*	summer	0.7113	148	0.80	0.01	131
R10121	R10121.O+P	*Equus sp.*	winter	0.7111	125	0.94	0.01	143
R10122	R10122.B	*Equus sp.*	summer	0.7107	108	0.82	0.00	42
R10122	R10122.K	*Equus sp.*	winter	0.7104	95	1.00	-	30
R10123	R10123.J	*Equus sp.*	summer	0.7099	143	1.07	-	35
R10123	R10123.N	*Equus sp.*	winter	0.7095	126	1.09	-	36
R10124	R10124.C	*Equus sp.*	summer	0.7107	152	1.06	-	37
R10124	R10124.K	*Equus sp.*	winter	0.7107	116	1.07	-	35
R10125	R10125.F+G	*Equus sp.*	winter	0.7090	221	0.90	-	57
R10125	R10125.I+J	*Equus sp.*	summer	0.7090	212	1.01	-	55
R10126	R10126.D	*Equus sp.*	summer	0.7095	129	1.30	-	15
R10126	R10126.J	*Equus sp.*	winter	0.7093	144	1.29	-	16
R10127	R10127.B	*Equus sp.*	summer	0.7113	113	1.17	0.02	58
R10127	R10127.F+G	*Equus sp.*	winter	0.7117	108	1.21	0.01	121
R10128	R10128.C	*Equus sp.*	summer	0.7107	121	0.99	-	36
R10128	R10128.F	*Equus sp.*	winter	0.7106	114	0.80	0.00	32
R10129	R10129.B	*Equus sp.*	summer	0.7111	114	0.99	-	19
R10129	R10129.J	*Equus sp.*	winter	0.7116	112	0.84	-	16
R10130	R10130.J	*Equus sp.*	summer	0.7112	124	0.96	-	13

SQUID	Sample ID	Taxon	Record type	$^{87}Sr/^{86}Sr$	Sr conc (ppm)	$\delta^{66}Zn$	SD	Zn conc (ppm)
R10130	R10130.Q	*Equus sp.*	winter	0.7113	108	0.93	-	14
R10131	R10131.F	*Equus sp.*	summer	0.7112	109	1.51	0.00	56
R10131	R10131.M	*Equus sp.*	winter	0.7120	108	1.12	0.00	87
R10132	R10132.F	*Equus sp.*	summer	0.7103	126	0.89	-	18
R10132	R10132.M	*Equus sp.*	winter	0.7100	131	0.79	-	15
R10141	R10141.D	*Equus sp.*	summer	0.7105	117	0.98	0.01	76
R10141	R10141.G	*Equus sp.*	winter	0.7105	124	1.00	0.01	79
R10142	R10142.I	*Equus sp.*	winter	0.7114	97	1.01	0.01	74
R10142	R10142.M	*Equus sp.*	summer	0.7115	90	1.03	0.01	69
R10148a	R10148a	*Cervid*	bulk	0.7122	136	0.77	0.02	38
R10148b	R10148b	*Rangifer tarandus*	bulk	0.7108	132	0.78	0.00	149
R10149a	R10149a	*Vulpes lagopus*	bulk	-	-	0.64	0.00	78
R10149b	R10149b	*Vulpes lagopus*	bulk	0.7112	110	0.34	0.00	235
R10150	R10150	*Coelodonta antiquitatis*	bulk	0.7103	60	0.77	0.01	43
R10151	R10151	*Cervus elaphus*	bulk	0.7089	154	1.10	0.01	74
R10152	R10152	*Cervid*	bulk	0.7107	119	0.91	0.00	77
R10156	R10156	*Crocuta spelaea*	bulk	0.7133	129	0.76	0.01	46
R10157	R10157	*Crocuta spelaea*	bulk	0.7131	115	0.70	0.01	105
R10158	R10158	*Crocuta spelaea*	bulk	0.7101	86	0.82	0.00	52
R10159	R10159	*Ursus spelaeus*	bulk	0.7099	125	0.88	0.00	40
R10160	R10160	*Ursus spelaeus*	bulk	0.7083	41	1.07	0.01	96
R10161	R10161	*Vulpes lagopus*	bulk	0.7103	82	0.54	-	103
R10162	R10162	*Coelodonta antiquitatis*	bulk	0.7106	156	0.57	0.00	83
R10163	R10163	*Cervus elaphus*	bulk	0.7117	98	0.82	0.00	96
R10164	R10164	*Vulpes lagopus*	bulk	0.7107	89	0.64	0.00	84
R10165	R10165	*Cervus elaphus*	bulk	0.7117	89	0.80	0.00	92

SQUID	Sample ID	Taxon	Record type	$^{87}Sr/^{86}Sr$	Sr conc (ppm)	$\delta^{66}Zn$	SD	Zn conc (ppm)
R10166	R10166	*Cervus elaphus*	bulk	0.7104	154	0.79	0.01	62
R10167	R10167	*Cervid*	bulk	0.7108	112	0.83	0.00	70
R10168	R10168	*Coelodonta antiquitatis*	bulk	0.7113	125	0.79	0.00	82
R10169	R10169	*Cervid*	bulk	0.7112	104	1.03	0.01	49
R10170	R10170	*carnivore small*	bulk	0.7110	116	0.35	0.02	227
R10171	R10171	*Ursus spelaeus*	bulk	0.7121	88	0.43	0.00	74
R10172	R10172	*Rangifer tarandus*	bulk	0.7099	103	1.01	0.00	92

Supplementary Table 6: Drinking water oxygen isotope values and palaeotemperature estimates for summer, mean annual, and winter for each time bin with corresponding compound estimation error. Number of data points can vary between different seasons of the same time bin, as oxygen isotope time series often include multiple or incomplete years and therefore can contain more summer than winter data points that are available for palaeotemperature conversion and vice versa. Seasonal amplitudes refer to summer-winter temperature differences and are calculated from summer and winter palaeotemperature estimates.

Age bin (cal BP)	Record type	$\delta^{18}O_{dw}$	$\delta^{18}O_{dw}$ error	T_{Air}(°C)	T_{Air} error	$N_{datapoints}$
36000 - 39000	summer	-9.8	0.8	12.8	1.8	11
42000 - 43000	summer	-7.2	1.1	18.4	2.5	5
43000 - 45000	summer	-11.2	1.5	9.8	3.2	3
45000 - 48000	summer	-9.2	1.0	14.1	2.3	6
36000 - 39000	mean annual	-13.0	0.8	1.7	1.5	15
42000 - 43000	mean annual	-12.4	1.2	2.8	2.2	5
43000 - 45000	mean annual	-15.7	1.5	-3.5	2.9	3
45000 - 48000	mean annual	-13.3	0.9	1.1	1.8	9
36000 - 39000	winter	-16.5	0.9	-9.4	1.8	11
42000 - 43000	winter	-16.3	1.5	-9.0	2.9	3
43000 - 45000	winter	-20.7	1.9	-17.3	3.6	2
45000 - 48000	winter	-17.0	1.3	-10.3	2.4	5
36000 - 39000	seasonal amplitude	6.7	1.2	22.2	2.6	-
42000 - 43000	seasonal amplitude	9.1	1.9	27.4	3.8	-
43000 - 45000	seasonal amplitude	9.5	2.4	27.1	4.9	-
45000 - 48000	seasonal amplitude	7.8	1.6	24.4	3.3	-

References

1. Hülle, W. *Die Ilsenhöhle unter Burg Ranis in Thüringen*. (G. Fischer, 1977).
2. Desbrosse, R. & Kozlowski, J. *Hommes et climats à l'âge du mammouth : Le paléolithique supérieur d'Eurasie centrale*. (FeniXX, 1988).
3. Flas, D. La transition du Paléolithique moyen au supérieur dans la plaine septentrionale de l'Europe. *Anthropologica & Praehistorica* **119**, 7–14 (2008).
4. M.Grünberg, J. New AMS Dates for Palaeolithic and Mesolithic Camp Sites and Single Finds in Saxony-Anhalt and Thuringia (Germany). *Proceedings of the Prehistoric Society* **72**, 95–112 (2006).
5. Mylopotamitaki, D. *et al.* *Homo sapiens* reached the higher latitudes of Europe by 45,000 years ago. *Nature* (accepted).
6. Smith, G. M. *et al.* The ecology, subsistence and diet of 45,000-year-old *Homo sapiens* at Ilsenhöhle in Ranis, Germany. *Nature Ecology and Evolution* (accepted).
7. Clark, I. D. & Fritz, P. *Environmental isotopes in hydrogeology*. 328 (Lewis Publishers, 1997).
8. Rozanski, K., Araguás-Araguás, L. & Gonfiantini, R. Isotopic patterns in modern global precipitation. *Climate change in Continental Isotopic Records* **78**, 1–36 (1993).
9. Müller, S., Stumpp, C., Sørensen, J. H. & Jessen, S. Spatiotemporal variation of stable isotopic composition in precipitation: Post-condensational effects in a humid area. *Hydrological Processes* **31**, 3146–3159 (2017).
10. Stumpp, C., Klaus, J. & Stichler, W. Analysis of long-term stable isotopic composition in German precipitation. *Journal of Hydrology* **517**, 351–361 (2014).
11. Dansgaard, W. Stable isotopes in precipitation. *Tellus* **16**, 436–468 (1964).
12. Kohn, M. J. & Welker, J. M. On the temperature correlation of δ^{18}O in modern precipitation. *Earth and Planetary Science Letters* **231**, 87–96 (2005).
13. Kohn, M. J., Schoeninger, M. J. & Valley, J. W. Herbivore tooth oxygen isotope compositions: Effects of diet and physiology. *Geochimica et Cosmochimica Acta* **60**, 3889–3896 (1996).
14. Luz, B., Kolodny, Y. & Horowitz, M. Fractionation of oxygen isotopes between mammalian bone-phosphate and environmental drinking water. *Geochimica et Cosmochimica Acta* **48**, 1689–1693 (1984).
15. Longinelli, A. Oxygen isotopes in mammal bone phosphate: A new tool for paleohydrological and paleoclimatological research? *Geochimica et Cosmochimica Acta* **48**, 385–390 (1984).
16. Iacumin, P., Bocherens, H., Mariotti, a. & Longinelli, A. Oxygen isotope analyses of co-existing carbonate and phosphate in biogenic apatite: a way to monitor diagenetic alteration of bone phosphate? *Earth and Planetary Science Letters* **142**, 1–6 (1996).
17. Gat, J. R. *Isotope Hydrology: A Study of the Water Cycle*. vol. 6 189 (Imperila College Press, 2010).
18. Fricke, H. C. & O'Neil, J. R. Inter- and intra-tooth variation in the oxygen isotope composition of mammalian tooth enamel phosphate: Implications for palaeoclimatological and palaeobiological research. *Palaeogeography, Palaeoclimatology, Palaeoecology* **126**, 91–99 (1996).
19. Fricke, H. C., Clyde, W. C. & O'Neil, J. R. Intra-tooth variations in δ^{18}O (PO_4) of mammalian tooth enamel as a record of seasonal variations in continental climate variables. *Geochimica et Cosmochimica Acta* **62**, 1839–1850 (1998).
20. Dean, M. C. Growth layers and incremental markings in hard tissues; a review of the literature and some preliminary observations about enamel structure in *Paranthropus boisei*. *Journal of Human Evolution* **16**, 157–172 (1987).
21. Hillson, S. *Teeth*. (Cambridge University Press, 2005).

22. Hoppe, K. A., Stover, S. M., Pascoe, J. R. & Amundson, R. Tooth enamel biomineralization in extant horses: Implications for isotopic microsampling. *Palaeogeography, Palaeoclimatology, Palaeoecology* **206**, 355–365 (2004).
23. Zazzo, A., Lécuyer, C. & Mariotti, A. Experimentally-controlled carbon and oxygen isotope exchange between bioapatites and water under inorganic and microbially-mediated conditions. *Geochimica et Cosmochimica Acta* **68**, 1–12 (2004).
24. Skrzypek, G., Sadler, R. & Wiśniewski, A. Reassessment of recommendations for processing mammal phosphate δ^{18}O data for paleotemperature reconstruction. *Palaeogeography, Palaeoclimatology, Palaeoecology* **446**, 162–167 (2016).
25. Tütken, T., Furrer, H. & Walter Vennemann, T. Stable isotope compositions of mammoth teeth from Niederweningen, Switzerland: Implications for the Late Pleistocene climate, environment, and diet. *Quaternary International* **164-165**, 139–150 (2007).
26. Pryor, A. J. E., Stevens, R. E., O'Connell, T. C. & Lister, J. R. Quantification and propagation of errors when converting vertebrate biomineral oxygen isotope data to temperature for palaeoclimate reconstruction. *Palaeogeography, Palaeoclimatology, Palaeoecology* **412**, 99–107 (2014).
27. Hoppe, K. A. Correlation between the oxygen isotope ratio of North American bison teeth and local waters: Implication for paleoclimatic reconstructions. *Earth and Planetary Science Letters* **244**, 408–417 (2006).
28. Rozanski, K., Araguás-Araguás, L. & Gonfiantini, R. Relation between long-term trends of oxygen-18 isotope composition of precipitation and climate. *Science* **258**, 981–985 (1992).
29. Arppe, L. M. & Karhu, J. A. Oxygen isotope values of precipitation and the thermal climate in Europe during the middle to late Weichselian ice age. *Quaternary Science Reviews* **29**, 1263–1275 (2010).
30. Rozanski, K. Deuterium and oxygen-18 in European groundwaters - Links to atmospheric circulation in the past. *Chemical Geology: Isotope Geoscience section* **52**, 349–363 (1985).
31. Zuber, A., Weise, S. M., Motyka, J., Osenbrück, K. & Różański, K. Age and flow pattern of groundwater in a Jurassic limestone aquifer and related Tertiary sands derived from combined isotope, noble gas and chemical data. *Journal of Hydrology* **286**, 87–112 (2004).
32. Kaspar, F., Kühl, N., Cubasch, U. & Litt, T. A model-data comparison of European temperatures in the Eemian interglacial. *Geophysical Research Letters* **32**, 1–5 (2005).
33. Arppe, L. M. & Karhu, J. A. Oxygen isotope values of precipitation and the thermal climate in Europe during the middle to late Weichselian ice age. *Quaternary Science Reviews* **29**, 1263–1275 (2010).
34. Schrag, D. P. *et al.* The oxygen isotopic composition of seawater during the Last Glacial Maximum. *Quaternary Science Reviews* **21**, 331–342 (2002).
35. Maloiy, G. M. O. Water metabolism of East African ruminants in arid and semi-arid regions. *Zeitschrift für Tierzüchtung und Züchtungsbiologie* **90**, 219–228 (1973).
36. Arias, R. A. & Mader, T. L. Environmental factors affecting daily water intake on cattle finished in feedlots. *Journal of Animal Science* **89**, 245–251 (2011).
37. McHugh, T. Social behavior of the American buffalo (Bison bison bison). *Zoologica* **43**, 1–40 (1958).
38. Winchester, C. F. & Morris, M. J. Water Intake Rates of Cattle. *Journal of Animal Science* **15**, 722–740 (1956).
39. Groenendyk, S., English, P. B. & Abetz, I. External balance of water and electrolytes in the horse. *Equine Veterinary Journal* **20**, 189–193 (1988).
40. Scheibe, K. M., Eichhorn, K., Kalz, B., Streich, W. J. & Scheibe, A. Water consumption and watering behavior of Przewalski horses (*Equus ferus przewalskii*) in a semireserve. *Zoo Biology: Published in affiliation with the American Zoo and Aquarium Association* **17**, 181–192 (1998).
41. Houpt, K. A., Eggleston, A., Kunkle, K. & Houpt, T. R. Effect of water restriction on equine behaviour and physiology. *Equine Veterinary Journal* **32**, 341–344 (2000).
42. Hinton, M. On the Watering of Horses: A Review. *Equine Veterinary Journal* **10**, 27–31 (1978).

43. Lécuyer, C., Hillaire-Marcel, C., Burke, A., Julien, M.-A. & Hélie, J.-F. Temperature and precipitation regime in LGM human refugia of southwestern Europe inferred from δ13C and δ18O of large mammal remains. *Quaternary Science Reviews* **255**, 106796 (2021).
44. Scherler, L., Tütken, T. & Becker, D. Carbon and oxygen stable isotope compositions of Late Pleistocene mammal teeth from dolines of Ajoie (Northwestern Switzerland). *Quaternary Research* **82**, 378–387 (2014).
45. Stevens, R. E., Balasse, M. & O'Connell, T. C. Intra-tooth oxygen isotope variation in a known population of red deer: Implications for past climate and seasonality reconstructions. *Palaeogeography, Palaeoclimatology, Palaeoecology* **301**, 64–74 (2011).
46. Fabre, M. *et al.* Late Pleistocene climatic change in the French Jura (Gigny) recorded in the δ18O of phosphate from ungulate tooth enamel. *Quaternary Research* **75**, 605–613 (2011).
47. Skrzypek, G., Winiewski, A. & Grierson, P. F. How cold was it for Neanderthals moving to Central Europe during warm phases of the last glaciation? *Quaternary Science Reviews* **30**, 481–487 (2011).
48. Stephan, E. Paläotemperaturbestimmungen anhand von Sauerstoffisotopenverhältnissen in Pferde und Rentierfunden aus der mittelpaläolithischen Fundstelle Salzgitter-Lebenstedt, Norddeutschland. in *Die Tierknochenfunde der mittelpaläolithischen Jägerstation von Salzgitter-Lebenstedt* (eds. Ludowici, B. & Pöppelmann, H.) (Braunschweigisches Landesmuseum, 2017).
49. Darling, W. G., Bath, A. H. & Talbot, J. C. The O & H stable isotopic composition of fresh waters in the British Isles. 2. Surface waters and groundwater. *Hydrology and Earth System Sciences* **7**, 183–195 (2003).
50. Halder, J., Terzer, S., Wassenaar, L. I., Araguás-Araguás, L. & Aggarwal, P. K. The Global Network of Isotopes in Rivers (GNIR): Integration of water isotopes in watershed observation and riverine research. *Hydrology and Earth System Sciences* **19**, 3419–3431 (2015).
51. Gonfiantini, R. Environmental isotopes in lake studies. in *Handbook of environmental isotope geochemistry; the terrestrial environment* (eds. Fritz, P. & Fontes, J.-C.) 113–168 (IAEA Vienna, 1986).
52. Helmens, K. F. The Last Interglacial–Glacial cycle (MIS 5–2) re-examined based on long proxy records from central and northern Europe. *Quaternary Science Reviews* **86**, 115–143 (2014).
53. Guse, B. *et al.* Analysing spatio-temporal process and parameter dynamics in models to characterise contrasting catchments. *Journal of Hydrology* **570**, 863–874 (2019).
54. Kern, O. A. *et al.* A near-continuous record of climate and ecosystem variability in Central Europe during the past 130 kyrs (Marine Isotope Stages 5–1) from Füramoos, southern Germany. *Quaternary Science Reviews* **284**, 107505 (2022).
55. Sirocko, F. *et al.* The ELSA-vegetation-stack: Reconstruction of landscape evolution zones (LEZ) from laminated Eifel maar sediments of the last 60,000 years. *Global and Planetary Change* **142**, 108–135 (2016).
56. Prud'homme, C. *et al.* Millennial-timescale quantitative estimates of climate dynamics in central Europe from earthworm calcite granules in loess deposits. *Communications Earth & Environment* **3**, 1–14 (2022).
57. Albert, J. & Sirocko, F. Evidence for an Extreme Cooling Event Prior to the Laschamp Geomagnetic Excursion in Eifel Maar Sediments. *Quaternary* **6**, 14 (2023).
58. Bentley, R. A. Strontium isotopes from the earth to the archaeological skeleton: A review. *Journal of Archaeological Method and Theory* **13**, 135–187 (2006).
59. Capo, R. C., Stewart, B. W. & Chadwick, O. A. Strontium isotopes as tracers of ecosystem processes: Theory and methods. *Geoderma* **82**, 197–225 (1998).
60. Dasch, E. J. Strontium isotopes in weathering profiles, deep-sea sediments, and sedimentary rocks. *Geochimica et Cosmochimica Acta* **33**, 1521–1552 (1969).
61. Steadman, L. T., Brudevold, F. & Smith, F. A. Distribution of strontium in teeth from different geographic areas. *The Journal of the American Dental Association* **57**, 340–344 (1958).

62. Flockhart, D. T. T., Kyser, T. K., Chipley, D., Miller, N. G. & Norris, D. R. Experimental evidence shows no fractionation of strontium isotopes ($^{87}Sr/^{86}Sr$) among soil, plants, and herbivores: Implications for tracking wildlife and forensic science. *Isotopes in Environmental and Health Studies* **51**, 372–381 (2015).

63. Britton, K. Multi-isotope analysis and the reconstruction of prey species and palaeoecology. (Durham University, 2010).

64. Pederzani, S. *et al.* Reconstructing Late Pleistocene paleoclimate at the scale of human behavior: an example from the Neandertal occupation of La Ferrassie (France). *Scientific Reports* **11**, 1419 (2021).

65. Maurer, A.-F. *et al.* Bioavailable $^{87}Sr/^{86}Sr$ in different environmental samples — Effects of anthropogenic contamination and implications for isoscapes in past migration studies. *Science of The Total Environment* **433**, 216–229 (2012).

66. Linnemann, U. & Romer, R. L. The Cadomian Orogeny in Saxo-Thuringia, Germany: Geochemical and Nd–Sr–Pb isotopic characterization of marginal basins with constraints to geotectonic setting and provenance. *Tectonophysics* **352**, 33–64 (2002).

67. Käßner, A., Kalapurakkal, H. T., Huber, B. & Tichomirowa, M. A New Water-Based 87Sr/86Sr Isoscape Map of Central and NE Germany, with Special Emphasis on Mountainous Regions. *Aquatic Geochemistry* **29**, 95–125 (2023).

68. Fortes, G. G. *et al.* Ancient DNA reveals differences in behaviour and sociality between brown bears and extinct cave bears. *Molecular Ecology* **25**, 4907–4918 (2016).

69. Gittleman, J. L. & Harvey, P. H. Carnivore home-range size, metabolic needs and ecology. *Behavioral Ecology and Sociobiology* **10**, 57–63 (1982).

70. Wißing, C. *et al.* Stable isotopes reveal patterns of diet and mobility in the last Neandertals and first modern humans in Europe. *Scientific Reports* **9**, 4433 (2019).

71. Pellegrini, M. *et al.* Faunal migration in late-glacial central Italy: Implications for human resource exploitation. *Rapid Communications in Mass Spectrometry* **22**, 1714–1726 (2008).

72. Pederzani, S. *et al.* Subarctic climate for the earliest Homo sapiens in Europe. *Science Advances* **7**, eabi4642 (2021).

73. Britton, K. *et al.* Multi-isotope zooarchaeological investigations at Abri du Maras: The paleoecological and paleoenvironmental context of Neanderthal subsistence strategies in the Rhône Valley during MIS 3. *Journal of Human Evolution* **174**, 103292 (2023).

74. Jaouen, K. & Pons, M.-L. Potential of non-traditional isotope studies for bioarchaeology. *Archaeological and Anthropological Sciences* **9**, 1389–1404 (2017).

75. Jaouen, K., Beasley, M., Schoeninger, M., Hublin, J.-J. & Richards, M. P. Zinc isotope ratios of bones and teeth as new dietary indicators: results from a modern food web (Koobi Fora, Kenya). *Scientific Reports* **6**, 26281 (2016).

76. Bourgon, N. *et al.* Zinc isotopes in Late Pleistocene fossil teeth from a Southeast Asian cave setting preserve paleodietary information. *Proceedings of the National Academy of Sciences* **117**, 4675–4681 (2020).

77. Bourgon, N. *et al.* Trophic ecology of a Late Pleistocene early modern human from tropical Southeast Asia inferred from zinc isotopes. *Journal of Human Evolution* **161**, 103075 (2021).

78. McCormack, J. *et al.* Zinc isotopes from archaeological bones provide reliable trophic level information for marine mammals. *Communications Biology* **4**, 1–11 (2021).

79. Jaouen, K., Szpak, P. & Richards, M. P. Zinc Isotope Ratios as Indicators of Diet and Trophic Level in Arctic Marine Mammals. *PLoS ONE* **11**, e0152299 (2016).

80. McCormack, J. *et al.* Trophic position of Otodus megalodon and great white sharks through time revealed by zinc isotopes. *Nature Communications* **13**, 2980 (2022).

81. Jaouen, K., Pons, M.-L. & Balter, V. Iron, copper and zinc isotopic fractionation up mammal trophic chains. *Earth and Planetary Science Letters* **374**, 164–172 (2013).

82. Vallee, B. L. & Falchuk, K. H. The biochemical basis of zinc physiology. *Physiological reviews* **73**, 79–118 (1993).
83. Loneragan, J. F. & Webb, M. J. Interactions Between Zinc and Other Nutrients Affecting the Growth of Plants. in *Zinc in soils and plants* (ed. Robson, A. D.) 119–134 (Springer Netherlands, 1993). doi:10.1007/978-94-011-0878-2_9.
84. Viers, J. *et al.* Evidence of Zn isotopic fractionation in a soil–plant system of a pristine tropical watershed (Nsimi, Cameroon). *Chemical Geology* **239**, 124–137 (2007).
85. Moynier, F. *et al.* Isotopic fractionation and transport mechanisms of Zn in plants. *Chemical Geology* **267**, 125–130 (2009).
86. Weiss, D. J. *et al.* Isotopic discrimination of zinc in higher plants. *New Phytologist* **165**, 703–710 (2005).
87. Viers, J. *et al.* Zn isotope fractionation in a pristine larch forest on permafrost-dominated soils in Central Siberia. *Geochemical Transactions* **16**, 3 (2015).
88. Moynier, F., Vance, D., Fujii, T. & Savage, P. The isotope geochemistry of zinc and copper. *Reviews in Mineralogy and Geochemistry* **82**, 543–600 (2017).
89. Cloquet, C., Carignan, J., Lehmann, M. F. & Vanhaecke, F. Variation in the isotopic composition of zinc in the natural environment and the use of zinc isotopes in biogeosciences: A review. *Analytical and Bioanalytical Chemistry* **390**, 451–463 (2008).
90. Pichat, S., Douchet, C. & Albarède, F. Zinc isotope variations in deep-sea carbonates from the eastern equatorial Pacific over the last 175 ka. *Earth and Planetary Science Letters* **210**, 167–178 (2003).
91. Balter, V. *et al.* Bodily variability of zinc natural isotope abundances in sheep. *Rapid Communications in Mass Spectrometry* **24**, 605–612 (2010).
92. Jaouen, K. *et al.* Zinc isotope variations in archeological human teeth (Lapa do Santo, Brazil) reveal dietary transitions in childhood and no contamination from gloves. *PLoS ONE* **15**, e0232379 (2020).
93. Jaouen, K. *et al.* A Neandertal dietary conundrum: Insights provided by tooth enamel Zn isotopes from Gabasa, Spain. *Proceedings of the National Academy of Sciences* **119**, e2109315119 (2022).
94. McArthur, J. M., Howarth, R. J. & Bailey, T. R. Strontium isotope stratigraphy: LOWESS version 3: Best fit to the marine Sr-isotope curve for 0-509 Ma and accompanying look-up table for deriving numerical age. *Journal of Geology* **109**, 155–170 (2001).
95. Lehmann, B. *et al.* Early Cambrian highly metalliferous black shale in South China: Cu and Zn isotopes and a short review of other non-traditional stable isotopes. *Mineralium Deposita* **57**, 1167–1187 (2022).
96. Romer, R. L. & Hahne, K. Life of the Rheic Ocean: Scrolling through the shale record. *Gondwana Research* **17**, 236–253 (2010).
97. Lécuyer, C. *et al.* Synthesis of In-House Produced Calibrated Silver Phosphate with a Large Range of Oxygen Isotope Compositions. *Geostandards and Geoanalytical Research* **43**, 681–688 (2019).
98. Johnson, C. M. & Fridrich, C. J. Non-monotonic chemical and O, Sr, Nd, and Pb isotope zonations and heterogeneity in the mafic- to silicic-composition magma chamber of the Grizzly Peak Tuff, Colorado. *Contributions to Mineralogy and Petrology* **105**, 677–690 (1990).
99. McCoy-West, A. J., Fitton, J. G., Pons, M.-L., Inglis, E. C. & Williams, H. M. The Fe and Zn isotope composition of deep mantle source regions: Insights from Baffin Island picrites. *Geochimica et Cosmochimica Acta* **238**, 542–562 (2018).
100. Fewlass, H. *et al.* Pretreatment and gaseous radiocarbon dating of 40–100 mg archaeological bone. *Scientific Reports* **9**, 5342 (2019).
101. Talamo, S., Fewlass, H., Maria, R. & Jaouen, K. 'Here we go again': The inspection of collagen extraction protocols for ^{14}C dating and palaeodietary analysis. *STAR: Science & Technology of Archaeological Research* **7**, 62–77 (2021).
102. Ramsey, C. B., Higham, T., Bowles, A. & Hedges, R. Improvements to the Pretreatment of Bone at Oxford. *Radiocarbon* **46**, 155–163 (2004).

103. Van Klinken, G. J. Bone collagen quality indicators for palaeodietary and radiocarbon measurements. *Journal of Archaeological Science* **26**, 687–695 (1999).

104. Kromer, B., Lindauer, S., Synal, H.-A. & Wacker, L. MAMS – A new AMS facility at the Curt-Engelhorn-Centre for Achaeometry, Mannheim, Germany. *Nuclear Instruments and Methods in Physics Research Section B: Beam Interactions with Materials and Atoms* **294**, 11–13 (2013).

105. Wacker, L., Němec, M. & Bourquin, J. A revolutionary graphitisation system: Fully automated, compact and simple. *Nuclear Instruments and Methods in Physics Research Section B: Beam Interactions with Materials and Atoms* **268**, 931–934 (2010).

106. Wacker, L. *et al.* MICADAS: Routine and High-Precision Radiocarbon Dating. *Radiocarbon* **52**, 252–262 (2010).

107. Wacker, L., Christl, M. & Synal, H.-A. Bats: A new tool for AMS data reduction. *Nuclear Instruments and Methods in Physics Research Section B: Beam Interactions with Materials and Atoms* **268**, 976–979 (2010).

108. Bronk Ramsey, C. Bayesian analysis of radiocarbon dates. Radiocarbon 51, 337e360. (2009).

109. Reimer, P. J. *et al.* The IntCal20 Northern Hemisphere radiocarbon age calibration curve (0–55 cal kBP). *Radiocarbon* **62**, 725–757 (2020).

110. Passey, B. H. *et al.* Inverse methods for estimating primary input signals from time-averaged isotope profiles. *Geochimica et Cosmochimica Acta* **69**, 4101–4116 (2005).

111. Fraser, D., Kim, S. L., Welker, J. M. & Clementz, M. T. Pronghorn (*Antilocapra americana*) enamel phosphate δ^{18}o values reflect climate seasonality: Implications for paleoclimate reconstruction. *Ecology and Evolution* **11**, 17005–17021 (2021).

112. Bendrey, R., Vella, D., Zazzo, A., Balasse, M. & Lepetz, S. Exponentially decreasing tooth growth rate in horse teeth: implications for isotopic analyses. *Archaeometry* **57**, 1104–1124 (2015).

113. Zazzo, A. *et al.* A refined sampling strategy for intra-tooth stable isotope analysis of mammalian enamel. *Geochimica et Cosmochimica Acta* **84**, 1–13 (2012).

114. Green, D. R. *et al.* Quantitative reconstruction of seasonality from stable isotopes in teeth. *Geochimica et Cosmochimica Acta* **235**, 483–504 (2018).

115. Kohn, M. J. Comment: Tooth enamel mineralization in ungulates: Implications for recovering a primary isotopic time-series, by B. H. Passey and T. E. Cerling (2002). *Geochimica et Cosmochimica Acta* **68**, 403–405 (2004).

116. Blumenthal, S. A. *et al.* Stable isotope time-series in mammalian teeth: In situ δ^{18}O from the innermost enamel layer. *Geochimica et Cosmochimica Acta* **124**, 223–236 (2014).

117. Delgado Huertas, A., Iacumin, P., Stenni, B., Sánchez Chillón, B. & Longinelli, A. Oxygen isotope variations of phosphate in mammalian bone and tooth enamel. *Geochimica et Cosmochimica Acta* **59**, 4299–4305 (1995).

118. Sánchez Chillón, B. *et al.* Oxygen isotopic composition of fossil equid tooth and bone phosphate: an archive of difficult interpretation. *Palaeogeography, Palaeoclimatology, Palaeoecology* **107**, 317–328 (1994).

119. Bryant, J. D., Luz, B. & Froelich, P. N. Oxygen isotopic composition of fossil horse tooth phosphate as a record of continental paleoclimate. *Palaeogeography, Palaeoclimatology, Palaeoecology* **107**, 303–316 (1994).

120. Blumenthal, S. A., Cerling, T. E., Smiley, T. M., Badgley, C. E. & Plummer, T. W. Isotopic records of climate seasonality in equid teeth. *Geochimica et Cosmochimica Acta* **260**, 329–348 (2019).

121. IAEA/WMO. Global Network of Isotopes in Precipitation. The GNIP Database. http://www.iaea.org/water. (2020).

122. R Core Team. R: A Language and Environment for Statistical Computing. (2020).

123. Allaire, J. J., Teague, C., Scheidegger, C., Xie, Y. & Dervieux, C. Quarto. (2022) doi:10.5281/zenodo.5960048.

124. Wilke, C. O. cowplot: Streamlined Plot Theme and Plot Annotations for 'ggplot2'. (2019).

125. Gohel, D. flextable: Functions for Tabular Reporting. (2020).

126. Clarke, E., Sherrill-Mix, S. & Dawson, C. *Ggbeeswarm: Categorical scatter (violin point) plots*. (2022).

127. Kassambara, A. *Ggcorrplot: Visualization of a correlation matrix using ggplot2*. (2022).

128. FC, M., Davis, T. L. & ggplot2 authors. *Ggpattern: ggplot2 pattern geoms*. (2022).

129. Kassambara, A. ggpubr: 'ggplot2' Based Publication Ready Plots. (2020).

130. Slowikowski, K. *Ggrepel: Automatically position non-overlapping text labels with ggplot2*. (2023).

131. Xie, Y. Knitr: A comprehensive tool for reproducible research in R. in *Implementing reproducible computational research* (eds. Stodden, V., Leisch, F. & Peng, R. D.) (Chapman; Hall/CRC, 2014).

132. Ooms, J. magick: Advanced Graphics and Image-Processing in R. (2020).

133. Mills, B. R. *MetBrewer: Color palettes inspired by works at the Metropolitan Museum of Art*. (2022).

134. Gohel, D. officer: Manipulation of Microsoft Word and PowerPoint Documents. (2020).

135. Wickham, H. & Seidel, D. *Scales: Scale functions for visualization*. (2022).

136. Wickham, H. *Tidyverse: Easily install and load the tidyverse*. (2023).

137. Zitzmann, A. Die geologische Übersichtskarte 1: 200 000–von der Karte bis zur Sachdatenbank. *Zeitschrift der Deutschen Geologischen Gesellschaft* **154**, 121–139 (2003).

138. BGR (Bundesanstalt für Geowissenschaften und Rohstoffe). Gruppen der Bodenausgangsgesteine in Deutschland 1:5.000.000 (BAG5000). (2007).

139. Bataille, C. P., Crowley, B. E., Wooller, M. J. & Bowen, G. J. Advances in global bioavailable strontium isoscapes. *Palaeogeography, Palaeoclimatology, Palaeoecology* **555**, 109849 (2020).
