# Supplementary material for: Stable isotopes show Homo sapiens dispersed into cold steppes ~45,000 years ago at Ilsenhöhle in Ranis, Germany
Source: Nat Ecol Evol. 2024 Jan 31;8(3):578–88. doi: 10.1038/s41559-023-02318-z (PMC10927559; doi:10.1038/s41559-023-02318-z)
Supplement: Supplementary file 2 — Reporting Summary [file 41559_2023_2318_MOESM2_ESM.pdf]

nature portfolio

Corresponding author(s): Sarah Pederzani

Last updated by author(s): 17.12.2023

Reporting Summary

Nature Portfolio wishes to improve the reproducibility of the work that we publish. This form provides structure for consistency and transparency in reporting. For further information on Nature Portfolio policies, see our Editorial Policies and the Editorial Policy Checklist.

Statistics

For all statistical analyses, confirm that the following items are present in the figure legend, table legend, main text, or Methods section.

n/a	Confirmed	
☐	☒	The exact sample size (*n*) for each experimental group/condition, given as a discrete number and unit of measurement
☐	☒	A statement on whether measurements were taken from distinct samples or whether the same sample was measured repeatedly
☐	☒	The statistical test(s) used AND whether they are one- or two-sided<br>*Only common tests should be described solely by name; describe more complex techniques in the Methods section.*
☒	☐	A description of all covariates tested
☐	☒	A description of any assumptions or corrections, such as tests of normality and adjustment for multiple comparisons
☐	☒	A full description of the statistical parameters including central tendency (e.g. means) or other basic estimates (e.g. regression coefficient) AND variation (e.g. standard deviation) or associated estimates of uncertainty (e.g. confidence intervals)
☐	☒	For null hypothesis testing, the test statistic (e.g. *F*, *t*, *r*) with confidence intervals, effect sizes, degrees of freedom and *P* value noted<br>*Give P values as exact values whenever suitable.*
☒	☐	For Bayesian analysis, information on the choice of priors and Markov chain Monte Carlo settings
☒	☐	For hierarchical and complex designs, identification of the appropriate level for tests and full reporting of outcomes
☐	☒	Estimates of effect sizes (e.g. Cohen's *d*, Pearson's *r*), indicating how they were calculated

Our web collection on statistics for biologists contains articles on many of the points above.

Software and code

Policy information about availability of computer code

Data collection	During IRMS data collection Isodat 3.0 was used.
Data analysis	Data analysis was conducted using R version 4.2.0. All analysis code is available at https://osf.io/wunfd/

For manuscripts utilizing custom algorithms or software that are central to the research but not yet described in published literature, software must be made available to editors and reviewers. We strongly encourage code deposition in a community repository (e.g. GitHub). See the Nature Portfolio guidelines for submitting code & software for further information.

Data

Policy information about availability of data

All manuscripts must include a data availability statement. This statement should provide the following information, where applicable:

- Accession codes, unique identifiers, or web links for publicly available datasets
- A description of any restrictions on data availability
- For clinical datasets or third party data, please ensure that the statement adheres to our policy

All data generated for this study, including measurements and data analysis results are available at https://osf.io/wunfd/

Research involving human participants, their data, or biological material

Policy information about studies with human participants or human data. See also policy information about sex, gender (identity/presentation), and sexual orientation and race, ethnicity and racism.

Reporting on sex and gender	N/A
Reporting on race, ethnicity, or other socially relevant groupings	N/A
Population characteristics	N/A
Recruitment	N/A
Ethics oversight	N/A

Note that full information on the approval of the study protocol must also be provided in the manuscript.

Field-specific reporting

Please select the one below that is the best fit for your research. If you are not sure, read the appropriate sections before making your selection.

☐ Life sciences ☐ Behavioural & social sciences ☒ Ecological, evolutionary & environmental sciences

For a reference copy of the document with all sections, see nature.com/documents/nr-reporting-summary-flat.pdf

Ecological, evolutionary & environmental sciences study design

All studies must disclose on these points even when the disclosure is negative.

Study description	New oxygen, carbon, nitrogen, zinc and strontium stable isotope data and radiocarbon dates of 16 sequentially sampled equid teeth; zinc and strontium stable isotope data of 24 teeth from various omnivore, herbivore and carnivore taxa to reconstruct climate and environments faced by H. sapiens groups during the Middle to Upper Palaeolithic transition at Ilsenhöhle in Ranis, Germany.
Research sample	Equid teeth were targeted for serial sampling as they have high-crowned teeth and are obligate drinkers that reflect oxygen isotopes of meteoric water. Additional teeth for a variety of carnivore, omnivore, and herbivore taxa were chosen for Zn and Sr analysis to explore feeding ecology across large mammals in the food web. Teeth were chosen to cover the lower part of the stratigraphic sequence, representing the MP/UP transition.
Sampling strategy	For equids, a sample size of >4 teeth per archaeological unit was used to guide sampling, as this has been shown to yield sufficiently precise palaeotemperature estimates (uncertainty of ~ 2-4 °C) from oxygen stable isotope measurements in European Palaeolithic palimpsest contexts (see Pryor et al., 2014 Palaeo3). Teeth from other taxa were chosen from a single archaeological unit, Layer IX, as this layer offers that largest faunal collection in the lower stratigraphic sequence. Sample sizes were constrained by availability, with an aim of 4-5 teeth per taxon and > 8 teeth per dietary group (carnivore, omnivore, herbivore).
Data collection	S. Pederzani collected equid tooth samples and conducted serial sampling, sample preparation and IRMS measurements for oxygen stable isotope analysis. H. Fewlass and S. Talamo conducted collagen extraction, carbon and nitrogen stable isotope analysis and radiocarbon dating on equid dentine and mandible bone samples. M. Trost collected tooth enamel samples from non-equid taxa and conducted sample preparation for zinc and strontium stable isotope analysis. N. Bourgon and J. McCormack conducted zinc and strontium isotope measurements.
Timing and spatial scale	Two equid tooth samples from the 2016-2022 excavation were recovered in 2019 and obtained in 2020 from the Thüringer Landesamt für Denkmalpflege und Archäologie, Weimar, Germany. No other suitable equid teeth were recovered from the 2021 or 2022 campaigns of these renewed excavations. All other tooth samples were obtained in 2018-2019 from the collection of the 1932-1938 excavation campaign housed at the Museum für Vorgeschichte, Halle (Saale), Germany. Teeth originate from a range of squares across the extent of the excavations and square and depth information is given in Supplementary Table 1.
Data exclusions	In few cases, individual oxygen stable isotope measurements (of triplicate analyses conducted for each sample) were excluded if predetermined IRMS quality control criteria of peak shape and the relationship of sample amount to peak area did not conform to good quality measurements. In these cases, oxygen stable isotope delta values represent the average of two, rather than the typical three measurements per sample.
Reproducibility	All stable isotope analyses (O, C, N, Zn, Sr) were repeated on at least a subset of samples (all samples in triplicate for oxygen, all samples in duplicate for carbon and nitrogen, a subset in duplicate for Zn and Sr isotope analysis) to determine analytical reproducibility. Details of analytical reproducibility are described in Supplementary Text 5 (Extended methods). Additionally, all code and data to reproduce the manuscript text, figures, tables, statistical analyses, inverse modelling and temperature estimation are supplied in an associated online repository.

Randomization	N/A
Blinding	N/A

Did the study involve field work? ☒ Yes ☐ No

Field work, collection and transport

Field conditions	Excavations at Ilsenhöhle in Ranis were conducted from 2016-2022. Two equid teeth used in this study were recovered in July/ August 2019.
Location	All specimens were recovered from Ilsenhöhle in Ranis, Germany (50°39.7563'N, 11°33.9139'E).
Access & import/export	Samples were obtained from the Thüringer Landesamt für Denkmalpflege und Archäologie (TLDA), Weimar, Germany and the Landesamt für Denkmalpflege und Archäologie Sachsen-Anhalt, Museum für Vorgeschichte (LDA), Halle (Saale), Germany. Sampling was conducted at the MPI-EVA, Leipzig, Germany without need for exporting. Permissions for destructive sampling were given by the LDA on 18.04.2018 (Nr. 14/2018) and by the TLDA on 26.11.2019 (Vorgangsnummer 16/116).
Disturbance	The samples were obtained from excavations of the archaeological site. The area of the renewed excavations was kept as small as possible to reach the lowest layers following safety measures of stepped excavation levels.

Reporting for specific materials, systems and methods

We require information from authors about some types of materials, experimental systems and methods used in many studies. Here, indicate whether each material, system or method listed is relevant to your study. If you are not sure if a list item applies to your research, read the appropriate section before selecting a response.

Materials & experimental systems

n/a	Involved in the study
☒	☐ Antibodies
☒	☐ Eukaryotic cell lines
☐	☒ Palaeontology and archaeology
☒	☐ Animals and other organisms
☒	☐ Clinical data
☒	☐ Dual use research of concern
☒	☐ Plants

Methods

n/a	Involved in the study
☒	☐ ChIP-seq
☒	☐ Flow cytometry
☒	☐ MRI-based neuroimaging

Palaeontology and Archaeology

Specimen provenance	Samples were obtained from the Thüringer Landesamt für Denkmalpflege und Archäologie (TLDA), Weimar, Germany and the Landesamt für Denkmalpflege und Archäologie Sachsen-Anhalt, Museum für Vorgeschichte (LDA), Halle (Saale), Germany. Sampling was conducted at the MPI-EVA, Leipzig, Germany without need for exporting. Permissions for destructive sampling were given by the LDA on 18.04.2018 (Nr. 14/2018) and by the TLDA on 26.11.2019 (Vorgangsnummer 16/116). Specimen IDs issued by the museums are reported for all specimens in Supplementary Table 1
Specimen deposition	All specimens have been returned to the LDA and the TLDA, where they are curated under museum authority.
Dating methods	16 equid samples collected for this study were pretreated and measured for 14C dating as part of this study. Collagen extraction and purification (including ultrafiltration) was carried out at the MPI-EVA, Leipzig using published protocols, which are described in the methods section and the Supplementary Extended methods. The suitability of collagen extracts for measurement was assessed based on coll % yield, elemental data (C%, N%, C:N). Quality criteria for all samples is included in the Supplementary Extended methods and in Supplementary Table 2. Samples were graphitised and measured with a MICADAS AMS at ETH-ZURICH and MAMS. Both uncalibrated and calibrated dates and laboratory codes are reported in Supplementary Table 2. Dates were calibrated using OxCal 4.3 using the IntCal20 data set.

☒ Tick this box to confirm that the raw and calibrated dates are available in the paper or in Supplementary Information.

Ethics oversight	Permissions for destructive sampling were given by the LDA by the TLDA, who are the relevant archaeological authorities regulating protection of archaeological finds in Thuringia and Saxony-Anhalt, Germany.

Note that full information on the approval of the study protocol must also be provided in the manuscript.
